# Long-Term Lead Exposure Since Adolescence Causes Proteomic and Morphological Alterations in the Cerebellum Associated with Motor Deficits in Adult Rats

**DOI:** 10.3390/ijms21103571

**Published:** 2020-05-18

**Authors:** Luana Ketlen Reis Leão, Leonardo Oliveira Bittencourt, Ana Carolina Oliveira, Priscila Cunha Nascimento, Giza Hellen Nonato Miranda, Railson Oliveira Ferreira, Mariane Nabiça, Kelly Dantas, Aline Dionizio, Sabrina Cartágenes, Marília Afonso Rabelo Buzalaf, Maria Elena Crespo-Lopez, Cristiane S F Maia, Rafael Rodrigues Lima

**Affiliations:** 1 Laboratory of Functional and Structural Biology, Institute of Biological Sciences, Federal University of Pará, Belém, Pará 66075-110, Brazil; luanakleao@gmail.com (L.K.R.L.); leo.bittencourt25@gmail.com (L.O.B.); anacarolina@ufpa.br (A.C.O.); priscilacunha.n28@gmail.com (P.C.N.); gizahellen@hotmail.com (G.H.N.M.); railson_91@yahoo.com (R.O.F.); 2 Laboratory of Applied Analytical Spectrometry, Institute of Exact and Natural Sciences, Federal University of Pará - Belém, Pará 66075-110, Brazil; mariane_gama@hotmail.com (M.N.); kdgfernandes@ufpa.br (K.D.); 3 Department of Biological Sciences, Bauru Dental School, University of São Paulo - Bauru, São Paulo 17012-901, Brazil; alinesdionizio@usp.br (A.D.); mbuzalaf@fob.usp.br (M.A.R.B.); 4 Laboratory of Inflammation and Behavior Pharmacology, Pharmacy Faculty, Institute of Health Sciences, Federal University of Pará - Belém, Pará 66075-110, Brazil; sabrina_decarvalho@yahoo.com.br (S.C.); crismaia@ufpa.br (C.S.F.M.); 5 Laboratory of Molecular Pharmacology, Institute of Biological Sciences, Federal University of Pará - Belém, Pará 66075-110, Brazil; maria.elena.crespo.lopez@gmail.com

**Keywords:** Lead, locomotion activity, morphological alterations, synaptic alteration, apoptosis

## Abstract

Lead (Pb) is an environmental contaminant that presents a high risk for human health. We aimed to investigate the possible alterations triggered by the exposure to Pb acetate for a long period in motor performance and the possible relationship with biochemical, proteomic and morphological alterations in the cerebellum of rats. Male Wistar rats were exposed for 55 days, at 50 mg/Kg of Pb acetate, and the control animals received distilled water. Open field (OF) and rotarod tests; biochemistry parameters (MDA and nitrite); staining/immunostaining of Purkinje cells (PC), mature neurons (MN), myelin sheath (MS) and synaptic vesicles (SYN) and proteomic profile were analyzed. Pb deposition on the cerebellum area and this study drove to exploratory and locomotion deficits and a decrease in the number of PC, MN, SYN and MS staining/immunostaining. The levels of MDA and nitrite remained unchanged. The proteomic profile showed alterations in proteins responsible for neurotransmitters release, as well as receptor function and second messengers signaling, and also proteins involved in the process of apoptosis. Thus, we conclude that the long-term exposure to low Pb dose promoted locomotion and histological tracings, associated with alterations in the process of cell signaling, as well as death by apoptosis.

## 1. Introduction

Lead (Pb) is a toxic metal and an environmental contaminant widely used in the production of pesticides and fertilizers, gasoline and pigments, batteries, cosmetics, metal products such as ammunition, welding, plumbing pipes, among others [1,2,3].

Pb levels in the environment have increased more than 1000-folds in the last three centuries as a result of anthropogenic activity [4]. Humans living near hazardous waste sites may be exposed to lead and chemicals that contain lead by breathing air, drinking water, eating foods, or swallowing dust or dirt that contain lead [4,5,6,7]. According to the World Health Organization (WHO), Pb is a high risk for human health, being able to reach and accumulate in different tissues and organs, such as the liver, kidneys, lungs, brain, spleen, muscles, and heart [4,7]. After distribution to the organs, the metal that is not stored in mineralized tissues leaves the body by urine or your feces [4]. The Pb also may replace calcium ions and alter cell signaling [8].

Following an episode of lead exposure on a scale of 30–80μg/dl Pb in the blood, the human may experience clinical symptoms such as abdominal pain, colic gastrointestinal pain, constipation and intestinal paralysis [9]. In addition, animal and in vitro studies highlight the hepato-carcinogenic role of Pb, the liver being the first organ to contact xenobiotics [9]. Until 1943, it was believed that people exposed to acute doses of Pb, without death, were able to fully recover. However, Needleman et al. [10], demonstrated the first scientific evidence of permanent behavioral and physiological deficits in children exposed to acute doses of Pb.

In the Central Nervous System (CNS), studies have shown deficits in learning ability, cognition and intellectual development in humans exposed to Pb during some period of life [11,12,13,14]. Furthermore, literature data indicate that low Pb concentrations are sufficient to alter biochemical processes, as well as induce non-specific disorders in brain functioning, such as decreased perception, visual, hearing and cognitive deficits [15,16]. Children exposed to acute doses of Pb (80 g/dL) presented formation of cerebral edema, seizure episodes and coma, besides inducing encephalopathy [17]. However, the relationship between exposure to Pb over time on the central nervous system, and its influence on the function and structure of the cerebellum have not been understood. 

The cerebellum is interconnected with the contralateral cerebrum primarily through two polysynaptic circuits [18,19,20,21]. Cerebellum is the major organ that plays an important function in the control of sensor locomotion system, locomotion coordination and locomotion cognition [22,23]. Moreover, converging evidence from functional imaging, tracing, and clinical studies have supported a role for the cerebellum in higher cognitive functions. Transneuronal tracing methods have delineated cerebellar connections with various non-locomotion cortical regions (e.g., prefrontal cortex) that could serve as neural substrates for contributions of the cerebellum to cognitive function [24,25].

The aim of this study was to analyze the long-term alterations due to the chronic exposure to Pb in motor performance and the possible relationship with biochemical, proteomic and morphological alterations in the cerebellum of rats.

## 2. Results

### 2.1. The Cerebellar Damage Caused by Pb Exposure is Associated with Increased Levels of the Metal in Cerebellar Parenchyma of Adult Rats

**Figure 1:**
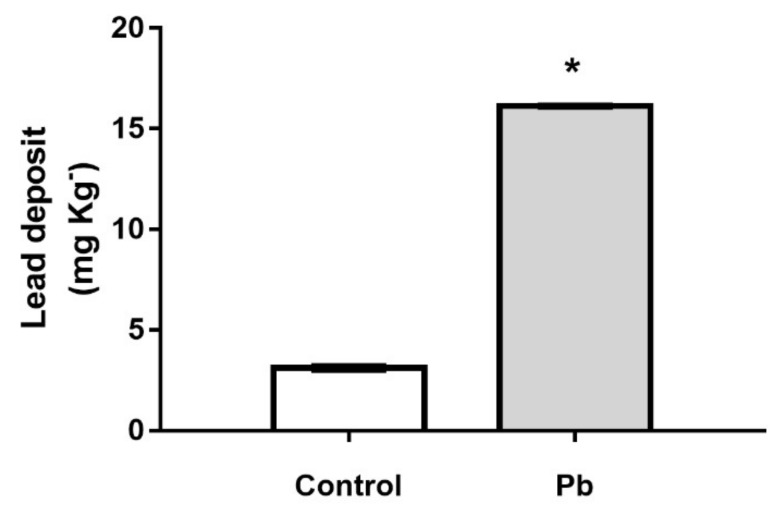
Effect of long-term exposure of young Wistar rats to 50 mg/Kg of Pb on lead deposition in cerebellum. The results are expressed as mean ± standard deviation. * Student’s t test, *p* < 0.0001.

After 55 days of exposure to lead acetate, the levels of Pb were significantly increased in cerebellar parenchyma in comparison to control group (Control: 3.12 ± 0.09 mg kg^−1^ vs Lead: 16.13 ± 0.03 mg kg^−1^; *p* < 0.0001) as showed in Figure 1.

### 2.2. Long-Term Exposure to Pb Since Adolescence Induced Cerebellar-Related Motor Impairments in the Adulthood.

**Figure 2:**
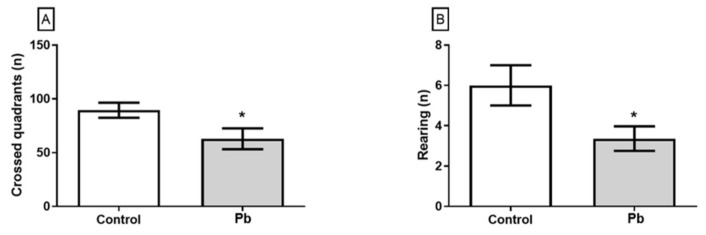
Effect of the long-term exposure of young Wistar rats to 50 mg/Kg of Pb on the exploratory activity in the open field test. In (**A**) crossed quadrants and (**B**) ‘rearing’. The results are expressed as mean ± standard error. * Student t test, *p* < 0.05.

Behavioral assessment of motor performance function showed that chronic exposure to Pb induced spontaneous motor performance deficits. Pb exposure reduced the number of crossed quadrants (Figure 2A) and rearing (Figure 2B) in open field test as showed in Figure 2. 

**Figure 3:**
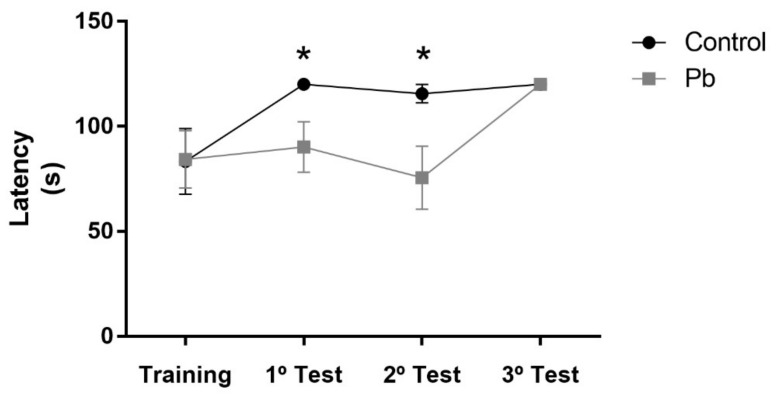
Effect of long-term exposure of young Wistar rats to 50 mg/Kg of Pb on locomotion activity in the Rotarod test. The results are expressed as mean ± standard error. * Lead vs. Control on different times (*one-way ANOVA with repeated measures*, *p* < 0.0001).

The training phase of rotarod test was performed equally by both groups (Figure 2). However, in the first and second exposure of the test section, Pb-exposed animals reduced the latency to the first fall, which reflects the poor performance on the coordination motor function. Pb-exposure animals only recovery the ability to perform properly the motor task on the third exposition to the rotating bar, which reveals the poor motor learning of the group (Figure 3). 

### 2.3. Long-Term Exposure to Pb Induced Cerebellar Tissue Damage of Rats

**Figure 4:**
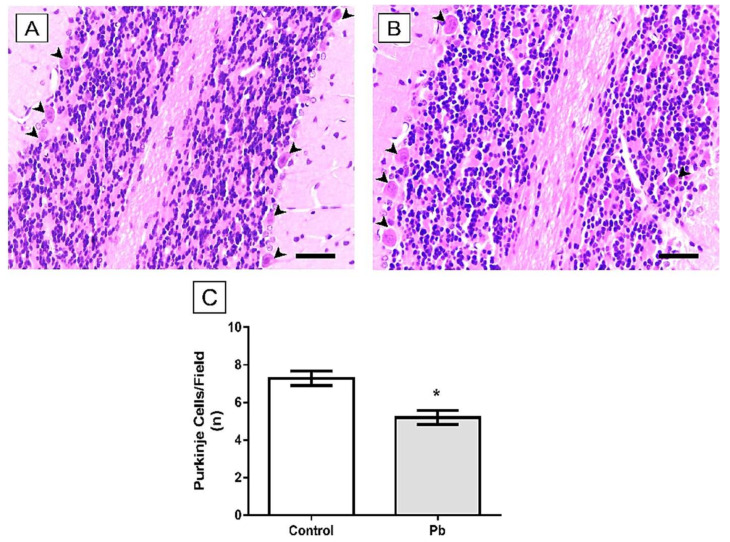
Effects of long-term exposure to Pb in the Purkinje cells (arrowhead) of cerebellum of Wistar rats. Sections were stained with hematoxylin and eosin (HE). (**A**) Control animal administered with distilled water and (**B**) animal exposed to lead. Results are expressed as mean ± standard error of (**C**) number of Purkinje cells. * *p* < 0.05 compared to control group (Student’s *t*-test). Scale bars: 30 μm.

In order to analyze whether exposure to Pb was able to induce alterations in cerebellar tissue morphology, we performed HE staining. Our results show that Pb acetate exposure reduces Purkinje cell population of rats Lead: 5.2 ± 0.38) when compared to the control group (Control group: 7.28 ± 0.37; *p* = 0.016; Figure 4).

**Figure 5:**
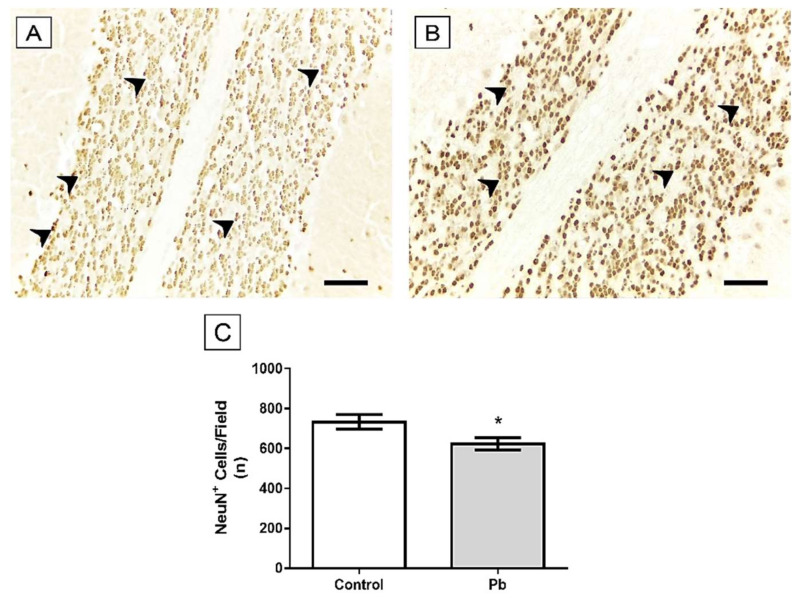
Effects of long-term exposure to Pb in the NeuN+ cells (arrowhead) in cerebellum of Wistar rats. Representative photomicrographs of the (**A**) Control group and (**B**) Lead group. Results are expressed as mean ± standard error of (**C**) NeuN+ cells density. * *p* < 0.05 compared to control group (Student’s t-test). Scale bars: 20 μm.

The analysis of the mature neuron population was performed through immunohistochemistry for NeuN. In the comparative analysis between the groups studied, NeuN+ cells per field of Pb group was decreased in relation to the animals’ control (Control group: 733.9 ± 35.43; Lead: 624.2 ± 30.3; *p* = 0.027, Figure 5). 

**Figure 6:**
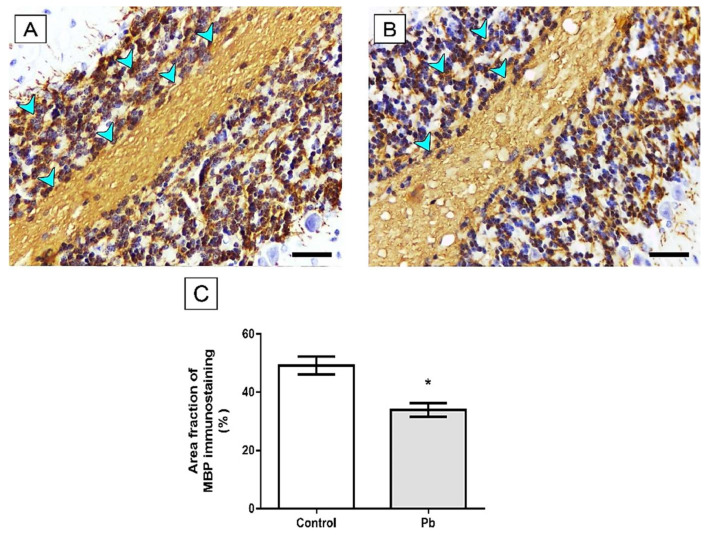
Effects of long-term exposure to Pb in Myelin basic protein (MBP) immunostaining (blue arrowhead) in cerebellum of Wistar rats. (**A**) Control group and (**B**) Lead group. The results are expressed as mean ± standard error of (**C**) area fraction percentage of immunostaining. * *p* < 0.05 compared to control group (Mann–Whitney test). Scale bars: 20 μm.

In addition to the morphological changes caused by exposure to lead, we observed that the proposed pattern was able to decrease the area fraction of MBP immunostaining in the group Pb (Lead: 33.86 ± 2.4) compared to the control group (Control group: 49.18 ± 3.08, *p* = 0.001), as seen in Figure 6.

**Figure 7:**
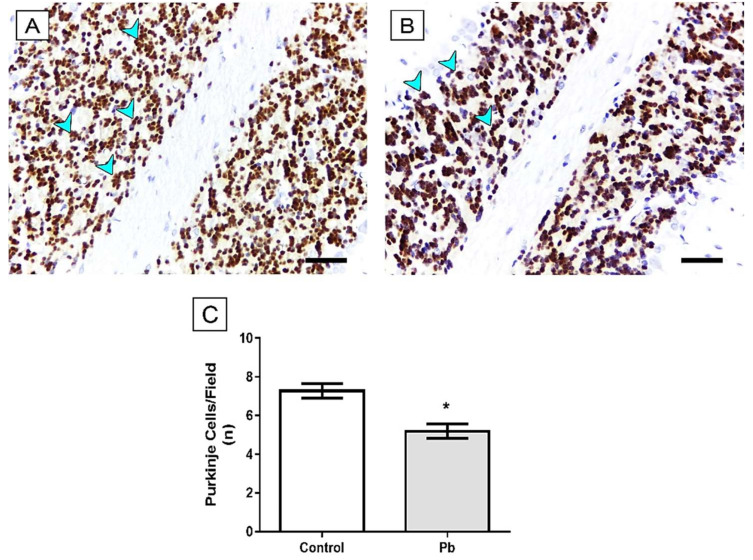
Effects of long-term exposure to Pb in synaptophysin (SYP) immunostaining (blue arrowhead) in cerebellum of Wistar rats. (**A**) Control group and (**B**) Lead group. The results are expressed as mean ± standard error of (**C**) area fraction percentage of immunostaining. * *p* < 0.05 compared to control group (Mann-Whitney test). Scale bars: 20 μm.

Furthermore, we also demonstrated a remarkable decrease in the area fraction of synaptophysin immunostaining (*p* = 0.001), indicating that our model of exposure to Pb causes damage to synaptic vesicles (Control group: 9.18 ± 0.36; Lead: 6.87 ± 0.54; Figure 7).

### 2.4. Long-Term Exposure to Pb is not Associated with Oxidative Stress Triggering 

**Figure 8:**
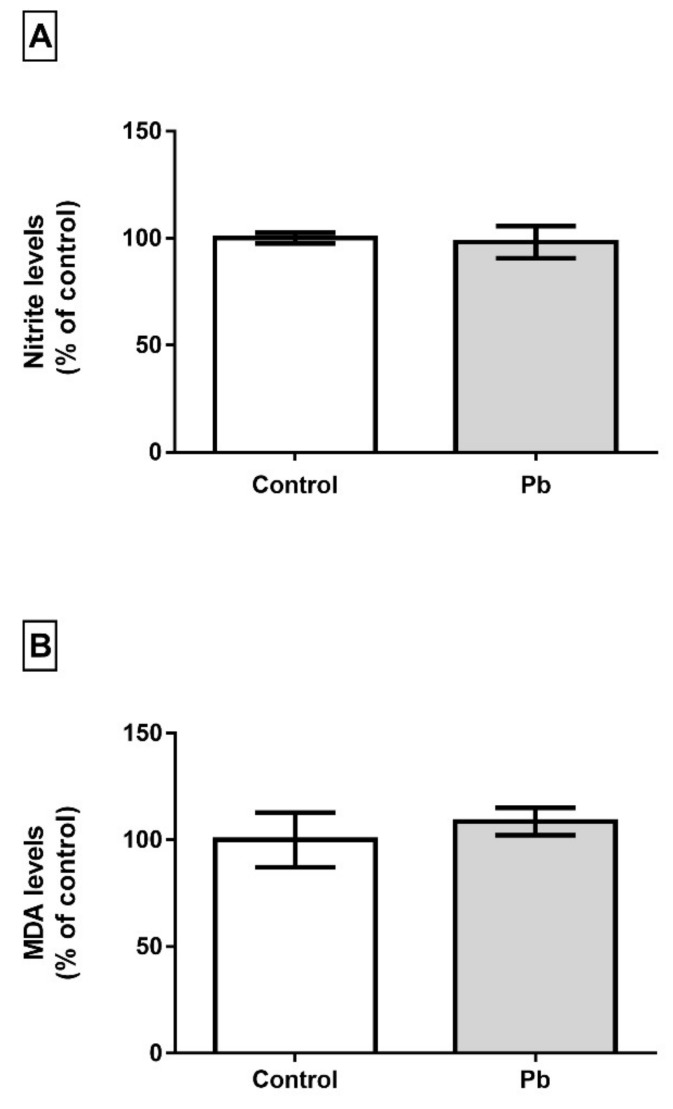
Effect of long-term exposure of young Wistar rats to 50 mg/Kg of Pb on oxidative biochemistry in cerebellum. In (**A**) nitrite levels, (**B**) MDA levels. The results are expressed as mean ± standard error after Student’s t-test analysis.

Although Pb levels were found increased in cerebellum, this event is not able to trigger oxidative biochemistry misbalance in cerebellum of adult rats in this model of exposure. The analysis of nitrite levels showed that long time exposure to Pb does not increase it levels in cerebellum (*p* = 0.3540) and LPO levels were not modulated either (*p* = 0.6141), as showed in Figure 8.

### 2.5. Long-Term Exposure to Pb Modulates Significantly the Cerebellum Proteomic Profile of Rats

The cerebellum proteome of rats long-term exposed to Pb showed 648 proteins with different status of expression, in which among them, 88 had their expression down-regulated and 142 down-regulated (Table S1), while 199 were found exclusively in the control group and 219 in the exposed group (Table S2). In Table 1, we highlighted proteins that underlie the other parameters analyzed in this study, such as oxidative stress, morphological and synaptic related proteins and cell homeostasis. The other proteins are available in the ESI (Tables S1 and S2).

**Figure 9:**
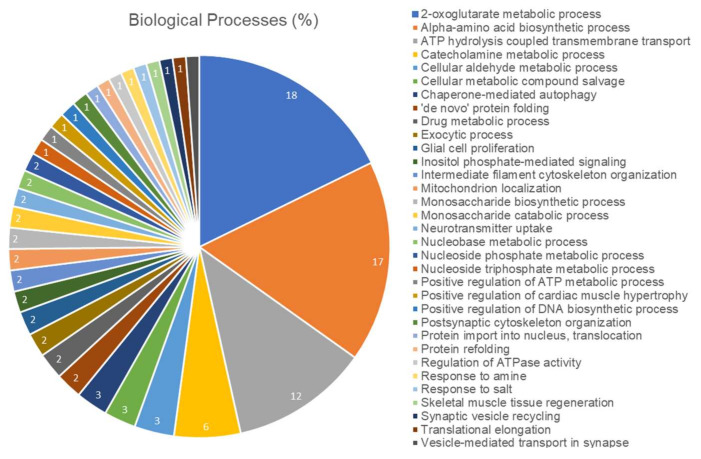
Functional distribution of proteins identified with differential expression in cerebellum of rats exposed to Pb vs. control group. Categories of proteins based on Gene Ontology annotation of biological process. Terms significance (Kappa Score = 0.4) and distribution according to percentage of number of genes. Proteins access number was provided by UNIPROT. The gene ontology was evaluated according to ClueGo^®^ plugin of Cytoscape^®^ software 3.6.1.

In addition, according to Figure 9, the proteome modulation of the cerebellum of rats exposed to Pb were associated to 33 groups of proteins with several biological processes according to Gene Ontology (GO) annotations. Among them, 2-Oxoglutarate metabolic process was the functional-related activity most impaired by Pb (18%), followed by alpha-amino acid biosynthetic process (17%), ATP hydrolysis coupled transmembrane transport (12%), cellular aldehyde metabolic process (6%), cellular metabolic compound salvage (3%) and others, that are associated to neurochemical activity, cellular components of cytoskeleton and neural cellular development.

**Figure 10:**
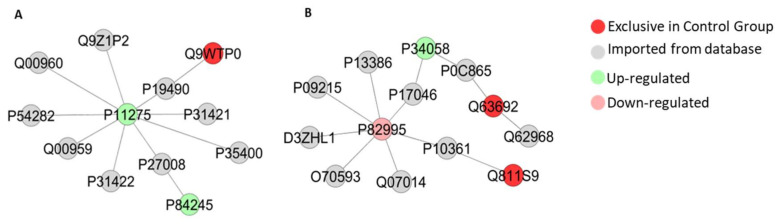
Sub-networks created by ClusterMarker app to determine the interaction among identified proteins of the cerebellum with different expression on exposed group vs. control group, named by its accession ID from Uniprot. In A: Calcium/calmodulin-dependent protein kinase type II subunit alpha (P11275), Glutamate receptor 1 (P19490), Band 4.1-like protein 1 (Q9WTP0), Metabotropic glutamate receptor 2 (P31421), Metabotropic glutamate receptor 7 (P35400), Poly [ADP-ribose] polymerase 1 (P27008), Histone H3.3 (P84245), Metabotropic glutamate receptor 3 (P31422), Glutamate receptor ionotropic, NMDA 2A (Q00959), Voltage-dependent P/Q-type calcium channel subunit alpha-1A (P54282), Glutamate receptor ionotropic, NMDA 2B (Q00960) and Alpha-actinin-1 (Q9Z1P2). In B: Heat shock protein HSP 90-alpha (P82995), Lysosome-associated membrane glycoprotein 2 (P17046), Heat shock protein HSP 90-beta (P34058), Mitogen-activated protein kinase 7 (P0C865), Hsp90 co-chaperone Cdc37 (Q63692), Sodium channel protein type 10 subunit alpha (Q62968), Cellular tumor antigen p53 (P10361), Guanine nucleotide-binding protein-like 3 (Q811S9), Tyrosine-protein kinase Lyn (Q07014), Small glutamine-rich tetratricopeptide repeat-containing protein alpha (O70593), Kinase suppressor of ras 1 (D3ZHL1), Protein kinase C delta type (P09215) and High affinity immunoglobulin epsilon receptor subunit beta (P13386).

**Figure 11:**
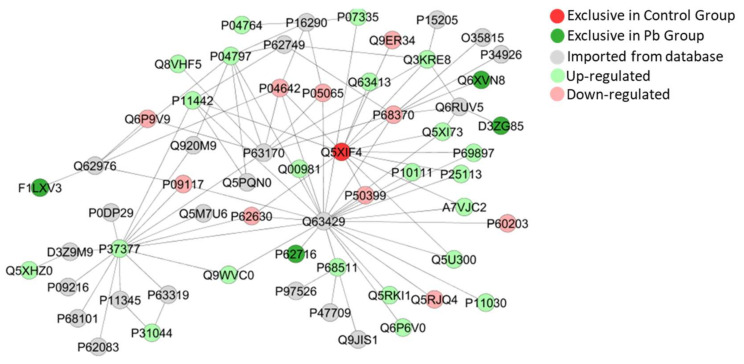
Sub-network created by ClusterMarker app to determine the interaction among identified proteins of the cerebellum with different expression on exposed group vs. control group, named by its accession ID from Uniprot. Serine/threonine kinase 26 (F1LXV3), Calcium-activated potassium channel subunit alpha-1 (Q62976), Tubulin alpha-1B chain (Q6P9V9), Clathrin heavy chain 1 (P11442), Citrate synthase, mitochondrial (Q8VHF5), Glyceraldehyde-3-phosphate dehydrogenase (P04797), Alpha-enolase (P04764), Hippocalcin-like protein 1 (P62749), Phosphoglycerate mutase 2 (P16290), Creatine kinase B-type (P07335), Aconitate hydratase, mitochondrial (Q9ER34), Microtubule-associated protein 1B (P15205), Tubulin beta-2B chain (Q3KRE8), Ataxin-3 (O35815), Microtubule-associated protein 1A (P34926), Microtubule-associated proteins 1A/1B light chain 3A (Q6XVN8), Ras-related C3 botulinum toxin substrate 1 (Q6RUV5), Cyclin-dependent kinase-like 5 (D3ZG85), Rho GDP-dissociation inhibitor 1 (Q5XI73), Tubulin beta-5 chain (P69897), Phosphoglycerate mutase 1 (P25113), Peptidyl-prolyl cis-trans isomerase A (P10111), Rab GDP dissociation inhibitor beta (P50399), Heterogeneous nuclear ribonucleoproteins A2/B1 (A7VJC2), Rab GDP dissociation inhibitor beta (P50399), Myelin proteolipid protein (P60203), Ubiquitin-like modifier-activating enzyme 1 (Q5U300), Acyl-CoA-binding protein (P11030), NAD-dependent protein deacetylase sirtuin-2 (Q5RJQ4), Glucose-6-phosphate isomerase (Q6P6V0), Eukaryotic initiation factor 4A-II (Q5RKI1), Regulating synaptic membrane exocytosis protein 2 (Q9JIS1), Rabphilin-3A (P47709), Neurofibromin (P97526), 14-3-3 protein eta (P68511), Serine/threonine-protein phosphatase 2A catalytic subunit beta isoform (P62716), Multidrug resistance protein 1 (Q63426), Septin-7 (Q9WVC0), Photosystem I reaction center subunit XI (P37277), Protein kinase C gamma type (P63319), Phosphatidylethanolamine-binding protein 1 (P31044), RAF proto-oncogene serine/threonine-protein kinase (P11345), 40S ribosomal protein S7 (P62083), Eukaryotic translation initiation factor 2 subunit 1 (P68101), Protein kinase C epsilon type (P09216), Heat shock protein 75 kDa, mitochondrial (Q5XHZ0), PTEN induced putative kinase 1 (Predicted) (D3Z9M9), Photosystem I reaction center subunit (XIP37277), Calmodulin-1 (P0DP29), Fructose-bisphosphate aldolase C (P09117), Actin-related protein 2 (Q5M7U6), Elongation factor 1-alpha 1 (P62630), Neurocalcin-delta (Q5PQN0), Dynein light chain 1, cytoplasmic (P63170), Ubiquitin carboxyl-terminal hydrolase isozyme L1 (Q00981), Small ubiquitin-related modifier 3 (Q5XIF4), L-lactate dehydrogenase A chain (P04642), Fructose-bisphosphate aldolase A (P05065), Spliceosome RNA helicase Ddx39b (Q63413) and Tubulin alpha-1A chain (P68370).

**Figure 12:**
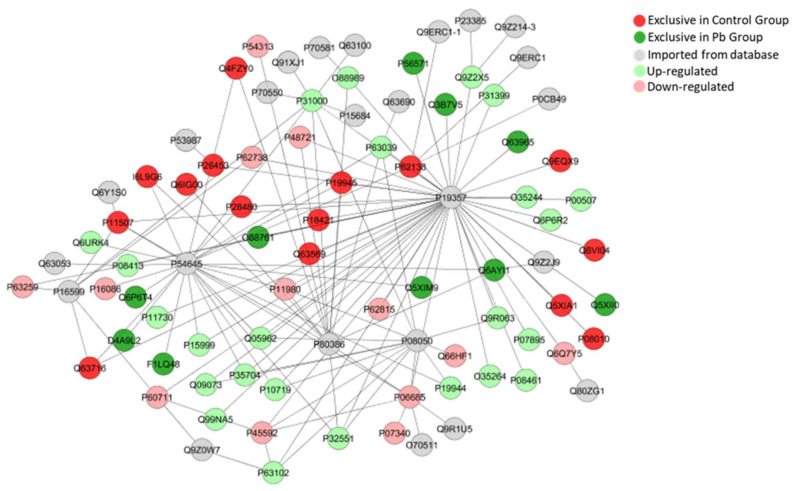
Sub-network created by ClusterMarker app to determine the interaction among identified proteins of the cerebellum with different expression on exposed group vs. control group, named by its accession ID from Uniprot. Solute carrier family 2, facilitated glucose transporter member 4 (P19357); Sideroflexin-1 (Q63965); Ubiquitin-conjugating enzyme E2 N (Q9EQX9); Peroxiredoxin-6 (O35244); Dihydrolipoyl dehydrogenase, mitochondrial (Q6P6R2); Aspartate aminotransferase, mitochondrial (P00507); Isoaspartyl peptidase/L-asparaginase (Q8VI04); Runt-related transcription factor 2 (Q9Z2J9); Nicalin (Q5XIA1); Mammalian ependymin-related protein 1 (Q5XII0); Glutathione S-transferase Mu 2 (P08010); Guanine nucleotide-binding protein subunit alpha-13 (Q6Q7Y5); Capsid protein (Q807G1); Dihydrolipoyl lysine-residue acetyltransferase component of pyruvate dehydrogenase complex, mitochondrial (P08461); Superoxide dismutase [Mn], mitochondrial (P07895); Peroxiredoxin-5, mitochondrial (Q9R063); DEAD (Asp-Glu-Ala-Asp) box polypeptide 5 (Q6AYI1); T-complex protein 1 subunit beta (Q5XIM9); Gap junction alpha-1 protein (P08050); NADH-ubiquinone oxidoreductase 75 kDa subunit, mitochondrial (Q66HF1); Platelet-activating factor acetylhydrolase IB subunit beta (O35264); 60S acidic ribosomal protein P1 (P19944); Sodium/potassium-transporting ATPase subunit alpha-1 (P06685); Sodium/potassium-transporting ATPase subunit beta-1 (P07340); Ankyrin-3 (O70511); Serine/threonine-protein kinase SIK1 (Q9R1U5); Cytochrome b-c1 complex subunit 2, mitochondrial (P32551); 14-3-3 protein zeta/delta (P63102); Cofilin-1 (P45592); ATP synthase subunit beta, mitochondrial (P10719); 5’-AMP-activated protein kinase subunit beta-1 (P80386); V-type proton ATPase subunit B, brain isoform (P62815); Chloride intracellular channel protein 4 (Q9Z0W7); Isocitrate dehydrogenase [NAD] subunit alpha, mitochondrial (Q99NA5); Peroxiredoxin-2 (P35704); ADP/ATP translocase 1 (Q05962); ADP/ATP translocase 2 (Q09073); ATP synthase subunit alpha, mitochondrial (P15999); Heterogeneous nuclear ribonucleoprotein L (F1LQ48); Actin, cytoplasmic 1 (P60711); Peroxiredoxin-1 (Q63716); RCG34610, isoform CRA_c (D4A9L2); Calcium/calmodulin-dependent protein kinase type II subunit gamma (P11730); Echinoderm microtubule-associated protein-like 2 (Q6P6T4); Spectrin alpha chain, non-erythrocytic 1 (P16086); Tumor necrosis factor (P16599); Actin, cytoplasmic 2 (P63259); Activity-regulated cytoskeleton-associated protein (Q63053); Heterogeneous nuclear ribonucleoprotein A3 (Q6URK4); Calcium/calmodulin-dependent protein kinase type II subunit beta (P08413); Sarcoplasmic/endoplasmic reticulum calcium ATPase 2 (P11507); Toll-like receptor 9 (Q6Y1S0); RCG31562, isoform CRA_c (I6L9G6); Keratin, type II cytoskeletal 4 (Q6IG00); 5’-AMP-activated protein kinase catalytic subunit alpha-1 (P54645); Pyruvate kinase PKM (P11980); 26S proteasome non-ATPase regulatory subunit 1 (O88761); T-complex protein 1 subunit alpha (P28480); Basigin (P26453); Monocarboxylate transporter 1 (P53987); Actin, aortic smooth muscle (P62738); EF-hand domain-containing protein D2 (Q4FZY0); Guanine nucleotide-binding protein G(I)/G(S)/G(T) subunit beta-2 (P54313); Ras-related protein Rab-8B (P70550); Beclin-1 (Q91XJ1); Nucleoporin p58/p45 (P70581); Cytoplasmic dynein 1 intermediate chain 1 (Q63100); Malate dehydrogenase, cytoplasmic (O88989); Aminopeptidase N (P15684); Apoptosis regulator BAX (Q63690); Rat tumor-associated aldehyde dehydrogenase (Q63039); Serine/threonine-protein phosphatase PP1-alpha catalytic subunit (P62138); Stress-70 protein, mitochondrial (P48721); 60S acidic ribosomal protein P0 (P19945); Proteasome subunit beta type-1 (P18421); 26S proteasome regulatory subunit 6A (Q63569); Vimentin (P31000); ES1 protein homolog, mitochondrial (P56571); Q9ERC1-1; Metabotropic glutamate receptor 1 (P23385); Q9Z214-3; Homer protein homolog 3 (Q9Z2X5); Unconventional myosin-XVI (Q9ERC1); ATP synthase subunit d, mitochondrial (P31399); RAB2B, member RAS oncogene Family (Q3B7V5); YLP motif-containing protein 1 (P0CB49).

The bioinformatic analyses of cerebellar proteome from rats exposed do Pb also presented subnetworks of protein-interactions as seen in Figure 10, Figure 11 and Figure 12, revealing interactions among vital proteins for cell metabolism and signaling functions, mainly associated to neural activities.

## 3. Discussion

In this study, for the first time, the cerebellar proteome imbalance induced by Pb long-term exposure, is associated with morphofunctional alterations. The evaluation of the proteomic profile of the cerebellum revealed important protein targets on Pb exposure, being associated with several biological processes responsible for organ maintenance, such as energetic metabolism, neural function and morphological maintenance. Still, we presented that this model of exposure is able of impairing spontaneous locomotion and motor coordination abilities, probably related to a synaptic dysfunction and myelin injury, and magnified by the reduction on cell density of neurons found in granule, molecular and Purkinje layers, which interestingly is not associated with oxidative/nitrosative stress triggering. 

In this work, the oral route was chosen as the route of administration of Pb. The exposure of human to Pb occurs mainly through gastrointestinal and respiratory tracts [26]. Serious episodes of human exposure to Pb in water have been recently detected, such as those at Flint (Michigan, USA) in 2014, called as “the major crisis of public health in the USA history”, or presently in many Canadian cities [27]. Once present in the bloodstream, Pb is easily diffused by the body, able to accumulate in several tissues, including the brain, due to its ability to replace Ca^2+^, making it able to cross the blood-brain barrier (BBB), as well as accumulate in astroglial cells (containing Pb-binding proteins), making it easy the induction of damage in these cells, hindering the formation of myelin sheath, both involved in the development of BBB [8,28,29,30,31].

Most studies with murine models use drinking water for exposure to Pb [8,27,30]. However, although the concentration of this metal is not variable, it is difficult to know exactly how much Pb is ingested during exposure. Therefore, the gavage process is more reliable [32]. After exposure of adolescent until adulthood Wistar rats to 50 mg/kg Pb acetate, tissue Pb levels were measured in cerebellar tissue, indicating Pb accumulation in this area. The Pb accumulation found with this exposure is similar to that described for exposed children by the CDC’s National Surveillance [33], and several studies have used animals models to mimic the infant exposure to Pb, highlighting the reflex over the developing CNS motor function [34,35,36].

The Pb induces damage mainly in the prefrontal cortex, cerebellum and hippocampus, when it is deposited in the encephalic region, affecting many biological activities at the molecular, cellular and intracellular levels, which may result in permanent functional alterations [37]. The cerebellum plays an important role in motor functions, being responsible basically, but not only, for modulation of locomotion performance, balance and fine-coordination [38]. The tests used in this study aimed to investigate specific parameters of cerebellar function, as spontaneous locomotion activity and equilibrium. Several studies showed that cerebellar damages may drive to poor locomotion performance; however, few studies characterize functional impairments related to cerebellum after exposure to Pb [39,40]. The results of our investigations of locomotion and coordination abilities showed that Pb exposure affects directly the capacity of vertical and horizontal spontaneous locomotion and reduced the latency time until the first fall, featuring cerebellar damage.

These behavioral alterations may be associated with histological alterations in the cerebellum. Our work has shown decreased numbers of Purkinje cells and mature neurons in the cerebellum, as well as decreased myelin sheath in the cerebellum and synaptic vesicles (Figure 4, Figure 5, Figure 6 and Figure 7). Morphological alterations of the brain [41] were observed after chronic exposure to Pb acetate. All these alterations in the composition of cerebellar tissue can directly compromise the functional activity of the animals after exposure to Pb [41]. 

The decrease in the number of mature neurons in the cerebellum as well as synaptic vesicles responsible for the release of neurotransmitters are fundamental for the physiological processes involved in the planning and execution of movements [42] and may be related to the Pb-induced locomotion alterations observed in this work (Figure 1 and Figure 2). A reduced number of cells was observed in both layers, Purkinje and molecular, not associated with oxidative/nitrosative stress. No difference was detected in the nitrite levels (an indirect marker of the production of nitric oxide) of the cerebellum after the chronic exposure to Pb, pointing to the absence of a significant generation of free radicals. This hypothesis is confirmed by the results of lipid peroxidation, one of the main deleterious consequences of oxidative stress, where no difference between control and exposed-group was detected. Similar results were observed by Dąbrowska-Bouta et al. [43], in which a lower dose of Pb was administered in drinking water for 3 months, and no significant LPO was detected after this Pb exposure. Although our study design did not show any redox imbalance, previous studies showed that Pb alters lipid metabolism, increasing the LPO, probably associated with others enzymes inhibition and increased flux of hydrogen peroxide and iron [44,45,46]. Moreover, the absence of increased of LPO and nitrite triggered due to possible protein rearrangements related to the oxidative biochemistry equilibrium, since when we evaluated the proteomic profile of the cerebellum, it was observed that ‘Superoxide dismutase [Mn], mitochondrial’ (P07895) and ‘Peroxiredoxin-5, mitochondrial’ (Q9R063), are up-regulated (Figure 12).

Furthermore, our bioinformatic analyses show that Pb exposure induced modulation of important proteins related to the apoptosis process in the cerebellum, such as ‘Calcium/calmodulin-dependent protein kinase type II subunit alpha’ (P11275) and ‘Cellular tumor antigen p53′ (P10361) (Figure 10), ‘Endoplasmic reticulum resident protein 29′ (P52555) and ‘Endoplasmic reticulum chaperone BiP’ (P06761) (Figure 11), and ‘Apoptosis regulator BAX’ (Q63690) (Figure 12). Excitotoxicity may be a causal factor for cell death caused by an imbalance on release and uptake of glutamate in synaptic cleft [47,48]. The clustered network in Figure 10A revealed interactions with Glutamate receptor 1 (P19490), Metabotropic glutamate receptor 2 (P31421), Metabotropic glutamate receptor 7 (P35400), Metabotropic glutamate receptor 3 (P31422), Glutamate receptor ionotropic, NMDA 2A (Q00959), Glutamate receptor ionotropic, NMDA 2B (Q00960) and Voltage-dependent P/Q-type calcium channel subunit alpha-1A (P54282), that interacted with other proteins found in our proteome. 

On the one hand, this is suggestive of a synaptic dysfunction, but on the other, it is directly associated with excitatory neurotransmission, suggesting that as well as other metals, Pb cell death and impairment of motor functions might be associated with excitotoxicity. In addition, this mechanism is generally associated with calcium imbalance in cells and in this way, several proteins of calcium transport and its function were found with different status of expression, as Calcium/calmodulin-dependent protein kinase II, beta, isoform CRA_a (G3V9G3; up-regulated), Calreticulin (P18418; up-regulated). Although we highlighted the role played by calcium in vesicle formation, circuits formation, calcium ions also play important roles in cell death mechanisms, as apoptosis (for review see Pinton et al., [49]). These proteins especially due to the intrinsic apoptosis pathway [50], intimately associated with mitochondrial failure, which may be suggested by the up-regulation of ATP synthase subunits (P19511, F1LP05, P15999, P10719, P31399 and P35434) in the Pb-exposed group, and observed in the clustered network on Figure 12.

For an efficient action potentiation (AP), we must consider two major questions: morphological integrity and communication efficiency [51,52]. From that, we elected two markers to investigate the Pb-induced cerebellar damage: MBP and Synaptophysin. The integrity of myelin sheath is fundamental for speed of transmission of action potentials along the axon and for that, MBP is a crucial and most abundant protein in myelin constitution [53,54]. Considering this, our immunohistochemical analyses showed a reduction in immunolabeling of anti-MBP, associated with the down-regulation of Myelin proteolipid protein (P60203), reported as an important protein for myelin maintenance [55,56]. Based on diseases characterized by demyelination like Multiple Sclerosis disease, which also affects cerebellar cortex and may drive do locomotion dysfunctions as loss of coordination and ataxia [57,58,59,60]. 

Furthermore, the second point that is involved in locomotion impairments after cerebellar damage, are neural cells failure and a consequent synaptic dysfunction [61]. Following this perspective, our proteomic approach also revealed that Clathrin heavy chain 1 (P11442) was found up-regulated, being related to vesicle clathrin-mediated endocytosis [62]. The cluster of protein interaction created by ClustMarker (Figure 12) demonstrated a strong association with proteins in vesicle trafficking, as GRIP1-associated protein 1(Q9JHZ4), found unique in exposed group and involved in the localization of recycling endosomes to dendritic spines [63]; Glutamate receptor-interacting protein 1 (P97879) and Glutamate receptor-interacting protein 2 (Q9WTW1), both integrants of myelin injury system were not detected, but interacted from databases with our study; and Vesicle-fusing ATPase (Q9QUL6), found up-regulated, which catalyzes the fusion of transport vesicles within the Golgi cisternae [64].

In this way, considering a large amount of effects caused by Pb exposure, our study concluded that the long-term exposure of young rats to 50 mg/Kg of lead acetate induces motor deficits and alterations in the cerebellar morphology. Moreover, Pb-exposure elicits proteomic profile modulation, not associated with oxidative biochemistry, but impairing several biological processes such as the release of neurotransmitters, postsynaptic receptors and second messengers, responsible for apoptosis. 

## 4. Materials and Methods 

### 4.1. Ethic Statement 

During the experimental period, the ethical standards for scientific research with laboratory animals were rigorously applied and this project was approved by the Ethics Committee from Federal University of Pará with protocol number 2237110716 26, approved on 07/28/2016.

### 4.2. Animals and Experimental Design 

Sixty male Wistar (Rattus norvegicus) rats of 40 postnatal days, weighing 150–160 g (*n* = 30/group) were treated by intragastric gavage with a daily dose of 50 mg/kg of lead acetate [Pb(C_2_H_3_O_2_)_2_] (Sigma-Aldrich, St. Louis, MO, USA) or distilled water (the same proportional volume to body weight) for 55 days. During the treatment period, water and food were provided ad libitum. This experimental paradigm was adapted from Gu et al. [65] which demonstrated accumulation in the cerebral tissue of 0.3 µg/g after exposure period of 50 mg/kg of lead acetate. 

### 4.3. Behavioral Assay

Twenty-four hours after the administration of the last dose of Pb, the animals were conducted to the assay room where sounds were attenuated, and the illumination was controlled in order to avoid stress during the habituation and test assays (1 h before the beginning of tests). Then, the animals were subjected to a behavioral test in which spontaneous locomotion, forced locomotion and balance assayed as follows:

#### 4.3.1. Open Field

The spontaneous locomotor activity of the experimental animals was evaluated by the open field test, adapted from Pandolfo et al. [66]. In this assay, the animals were positioned in the center of a wooden arena (100 × 100 × 40 cm), divided into 25 quadrants, in which free exploitation was permitted for 300 s. All sessions were videotaped and subsequently analyzed. The parameters evaluated were vertical exploration (i.e., number of times the animal was supported on the hind legs—‘rearing’), and horizontal exploration (i.e., number of quadrants crossed). After the open field test, animals were submitted to forced locomotion in gyratory cylinder (Rota-rod).

#### 4.3.2. Forced Locomotion in Gyratory Cylinder (Rota-rod)

The locomotor evaluation was performed in a rotating cylinder (Rota-rod), an automated device (Insight^®^ Brazil, EFF-411). The apparatus consists of an acrylic box with a cylinder with 8 cm of diameter, installed transversely 20 cm from the ground and is maintained in rotation by a motor. The box is divided into 4 bays, approximately 10 cm wide, allowing the analysis of 4 animals simultaneously. For the test, each animal was placed on the already moving cylinder and the time the animals were able to equilibrate until the first fall (latency) was recorded. In the fall, the timer that checked the length of stay on the cylinder was automatically stopped, since the equipment has a system installed in the ground of each bay that detects the impact of the fall. In the test training, animals were placed on the spin axis for a period of 120 s at 8 revolutions per minute (RPM). When the animals felt they were repositioned on the rotating axis as many times as were necessary until the stipulated time for this phase (120 s). After the training, the test was performed in three exposures of 120 s. The latency time, i.e., until the first fall (adapted from [67]) was recorded, as well as the number of falls.

### 4.4. Fresh Samples Collection 

After performing the behavioral assessment, 10 of the total number of animals of each group was anesthetized with a solution of ketamine hydrochloride (90 mg/kg, i.p.) and xylazine hydrochloride (10 mg/kg, i.p.) and then euthanized. The cerebellums were collected after craniotomy, followed by freezing in liquid nitrogen and stored at – 80 °C. These samples were posteriorly used for the analysis of lead and the biochemical (LPO and nitrite) and proteomic measurements.

### 4.5. Pb Levels Quantification

To quantify the Pb levels, the samples of the cerebellums were initially lyophilized individually with a L 101 lyophilizer (Liotop, São Carlos, Brazil). After lyophilization, a pool of samples was performed for each group. The mass of the samples was digested in 4.0 mL of HNO3 (14 mol/L), 2.0 mL of H_2_O_2_ (35% *w*/*w*) and 2.0 mL of ultrapure H_2_O in a microwave oven with cavity Start E (Milestone, Sorisole, Italy) at a temperature of 180 °C for 1 h and 15 min. After digestion, the solutions were transferred to volumetric flasks and filled to the final volume of 40 mL with ultrapure water. For determination of Pb, the samples were diluted to final acidity of 5.0%. An analytical curve of 2.0, 4.0, 6.0, 8.0 and 10.0 mg/L Pb was constructed for Pb determination in the samples using a microwave-induced plasma–optical emission spectrometer (Model 4100 Thermo Scientific, Waltham, MA, USA) (MIPOES). The wavelength used in the determination of lead was 405.781 nm. In order to evaluate the accuracy of the analysis, after samples reading, were added to the digested samples 3.5, 4.5 and 5.5 mg/L of standard Pb and then determined by MIPOES. The limits of detection and quantification were 0.57 mg/kg and 1.89 mg/kg. 

### 4.6. Oxidative Biochemistry Analyses

The samples of the cerebellum were thawed on ice, resuspended in Tris-HCl buffer (20 mM, pH 7.4) with an approximate concentration of 1 g/mL, sonically disaggregated and after the samples were separated into two aliquots. To analyze nitrite levels, the aliquots were centrifuged at 14,000 rpm for 10 min, while for lipid peroxidation assay, the samples were centrifuged at 5.600 rpm for 10 min, both at 4 °C. Only the supernatants were used for the analyses and for further protein concentration measurement for normalization, we performed Bradford’s method [68].

#### 4.6.1. Assay of Nitrite Levels

Nitrite levels were determined by Griess reaction with the Griess reagent (naphthyl-ethylenediamine 0.1% and sulfanilamide 1% in phosphoric acid 5%, 1:1) [69]. This reaction generates azoic compounds with a bluish coloration, proportionally to the nitrite concentration present in the sample. In a 96-well microplate, 100 µL of sample or standard nitrite solution and 100 µL of Griess reagent were incubated for 20 min at room temperature. Absorbance (λ = 570nm) was registered and the results were expressed as percentage of the control, after correction for protein concentration.

#### 4.6.2. Analysis of Lipid Peroxidation (LPO) by Malondialdehyde (MDA) levels

The levels of LPO were assayed by the method proposed by Esterbauer and Cheeseman [70]. Briefly, 325 μL of 10.3 mM N-methyl-2- phenylindole diluted in methanol (1:3) and 75 μL of methanesulfonic acid were added to 100 μL of standard MDA solutions or samples in a 96-well microplate. This mixture was heated at 45 °C for 40 min. Absorbance was then registered (λ = 570 nm) and the results are expressed as percentage of the control, after correction for protein concentration.

### 4.7. Proteomic Approach

#### 4.7.1. Proteomic Analysis: Preparation of the Samples

All proteomic analyses were performed according to protocols previously described by our group [71,72]. Firstly, the cerebellums from two animals were pooled and the analyses were carried out in triplicate. After pooled the samples, they were homogenized in liquid nitrogen by a cryogenic mill, followed by protein extraction with lysis buffer containing 7 M urea, 2 M thiourea and 40 mM dithiothreitol (DTT) diluted in ammonium bicarbonate (AmBic, 50mM) solution, under constant shaking at 4 °C. After extraction, the samples were centrifuged at 14,000 rpm for 30 min at 4 °C, in order to collect the supernatant and to quantify the amount of proteins by Bradford’s method [68] and to collect 50 μg of protein, which was filled with AmBic until the final volume of 50 μL (1 μg/μL). For each sample, 10μL of 50mM AmBic and 25 μL of 0.2% RapiGEST™ (Waters Co., Manchester, UK) were added and incubated at 37 °C for 30 min. After, the samples were reduced with 5 mM DTT at 37 °C for 40 min, followed by alkylated with 10 mM iodoacetamide (IAA) and incubation for 30 min at room temperature and absence of light. After, were digested with 2% (*w*/*w*) trypsin for 14 h at 37 °C with subsequent addition of 10 µL of 5% trifluoroacetic acid and incubation for 90 min at 37 ºC and subsequent centrifugation at 14,000 rpm for 30 min at 6 °C. After centrifugation, the supernatants were collected and purified using C18 Spin columns (Pierce™). Samples were resuspended in the solution containing 3% acetonitrile and 0.1% formic acid to be submitted to Mass Spectrometry. 

#### 4.7.2. Mass Spectrometry analyses

The reading and identification of the peptides were performed on a nanoAcquity UPLC-Xevo QTof MS system (Waters, Manchester, UK), using the Protein Lynx Global Server (PLGS), as previously described by Lima-Leite et al. [73]. PLGS software, applying the Monte-Carlo algorithm, was used to obtain the difference of protein expression between the groups, considering *p* < 0.05 for down-regulated proteins and p > 0.95 for up-regulated proteins. The database used for protein identification was the Rattus norvegicus (reviewed only, UniProtKB/Swiss-Prot) download on June 2019 from UniProtKB (http://www.uniprot.org/). After, the proteins identified were analyzed by a bioinformatic approach using Cytoscape 3.6.1 (Java^®^) with ClusterMarker plugin for protein-interaction network, and for determination of biological processes groups we used ClueGO plugin [74].

### 4.8. Perfusion and Histological Procedures

To evaluate morphological changes in the cerebellum, 7 animals of each group were anesthetized with a solution of ketamine hydrochloride (90 mg/kg i.p.) and xylazine hydrochloride (10 mg/kg, i.p.) and perfused through the left ventricle of the heart with solution heparinized 0.9% saline, followed by 4% paraformaldehyde. The samples were post-fixed in Bouin solution for 6 h and then immersed in 70%, 80%, 90%, absolute I, absolute II, xylol I and xylol II alcohol followed by paraplast (McCormick, Baltimore, MD, USA) embedding. After inclusion, the blocks were sectioned by a microtome to obtain sections with 5 μm of thickness.

#### 4.8.1. Purkinje Cell Counting 

For evaluation of Purkinje cell density, the sections obtained by microtomy were stained by routine Hematoxylin-Eosin (HE) and then analyzed by light microscopy (Nikon Eclipse E200) with a 1 mm^2^ grid attached to the ocular and using objective lens with 40× of magnification. At least 3 fields in the cerebellum per section and 3 sections per animal of each group were analyzed and counted the number of Purkinje neurons per field [75].

#### 4.8.2. Immunohistochemical Assays

For immunohistochemical analyses, the slides with sections were dewaxed and rehydrated by heat, xylene and hydroalcoholic solutions and immersed in PBS for 3 min before incubation in citrate buffer at 70 °C for 25 min. After antigen retrieval, they were immersed in PBS for 10 min and immersed in methanol-hydrogen peroxide solution (3:100, *v*/*v*) for endogenous peroxidase inhibition. Anti-NeuN (1:100, Chemicon), Anti-Myelin Basic Protein (MBP) (1:250, Promega) and Anti-Synaptophysin (1:1000, Wako) antibodies for immunolabeling of neurons, as well as MPB and synaptic vesicles, respectively, as previously established by our group were applied [22,75,76]. Revelation was proceeded by 3,3′-diaminobenzidine solution in PBS; sections immunolabeled with anti-MBP and anti-Synaptophysin were counter stained with Mayer’s hematoxylin. All slides were dehydrated and mounted with coverslip and Entellan^®^ (Merck, Darmstadt, Germany). 

#### 4.8.3. Quantitative Analyses of Immunohistochemistry Assays 

The positive cells for anti-NeuN immunostaining were analyzed by light microscopy (Nikon Eclipse E200) with a 1 mm^2^ attached to the ocular and using objective lens of 40x, evaluating the density of anti-NeuN+ cells. For analyses of anti-MBP and anti-Synaptophysin immunostaining, we first obtained the photomicrographs using the microscope Nikon Eclipse E500 with Moticam 2500^®^ attached to it. The photomicrographs were analyzed using ImageJ software with Color Deconvolution plugin according to previous works from our group [75,76], which extracted the fraction of area immunolabeled by the antibodies and revealed by DAB. The results are expressed as fraction of area (%) immunolabeled in comparison to the total area of section captured by the camera system.

### 4.9. Statistical Analyses 

For the analysis of the Gaussian distribution of the data, the Shapiro-Wilk normality test was performed. Differences between the two groups (control and exposed) were analyzed with the Student’s t-test and the Mann–Whitney test, for Gaussian and non-Gaussian data, respectively. Data of rotarod were tested with one-way ANOVA with repeated measures. The level of significance was set at *p* < 0.05 for all analyses. Power calculations for the difference between two means was done by using OpenEpi (Version 2.3.1), with type I error 5% and power of 80%. 

## Figures and Tables

**Table 1:** Highlighted proteins found in the proteome of rats cerebellum exposed to lead acetate in comparison to control.

Accesion ID ^a^	Protein Description	Score	Fold Change
P60203	Myelin proteolipid protein	794.51	−0.92
P19511	ATP synthase F(0) complex subunit B1, mitochondrial	212.89	−0.44
P11442	Clathrin heavy chain 1	174.43	1.06
P06761	Endoplasmic reticulum chaperone BiP	453.96	1.11
F1LP05	ATP synthase subunit alpha	2920.7	1.12
P15999	ATP synthase subunit alpha, mitochondrial	3053	1.12
P31399	ATP synthase subunit d, mitochondrial	665.55	1.12
P07895	Superoxide dismutase [Mn], mitochondrial	333.18	1.12
Q9R063	Peroxiredoxin-5, mitochondrial	596.72	1.15
P18418	Calreticulin	135.67	1.16
P35434	ATP synthase subunit delta, mitochondrial	314.76	1.17
P10719	ATP synthase subunit beta, mitochondrial	8483	1.20
Q9QUL6	Vesicle-fusing ATPase	130.82	1.25
G3V9G3	Calcium/calmodulin-dependent protein kinase II, beta, isoform CRA_a	317.64	1.52
G3V9G3	Calcium/calmodulin-dependent protein kinase II, beta, isoform CRA_a	317.64	1.52
P11275	Calcium/calmodulin-dependent protein kinase type II subunit alpha	164.49	1.65
Q9JHZ4	GRIP1-associated protein 1	30,243	Pb
	+631 proteins with different status of regulation		

^a^ Accesion ID from uniprot.org database; Signs of - and + represent down-regulation and up-regulation, respectively, in Pb group when compared to control group; Pb means that the protein was exclusively expressed in exposed group.

## Supplementary Materials

Supplementary materials can be found at https://www.mdpi.com/1422-0067/21/10/3571/s1.

## Abbreviations

AP	Action potentiation
BBB	Blood-brain barrier
CNS	Central Nervous System
GO	Gene Ontology
HE	Hematoxylin-Eosin
MBP	Myelin basic protein
MDA	Malondialdehyde
MDPI	Multidisciplinary Digital Publishing Institute
MS	Myelin sheath
OF	Open field
Pb	Lead
PC	Purkinje cells
SYN	Synaptic vesicles
WHO	World Health Organization
MDPI	Multidisciplinary Digital Publishing Institute
Pb	Lead
