# Supplementary material for: Long-Term Lead Exposure Since Adolescence Causes Proteomic and Morphological Alterations in the Cerebellum Associated with Motor Deficits in Adult Rats
Source: Int J Mol Sci. 2020 May 18;21(10):3571. doi: 10.3390/ijms21103571 (PMC7279001; doi:10.3390/ijms21103571)
Supplement: Supplementary file 1 [file ijms-21-03571-s001.pdf]

Table S1. Global proteomic profile of rats' cerebellum exposed or not to lead acetate. List of proteins with differential regulation in comparison to control group.

[a]Accession ID	Protein name description	PLGS Score	Fold Change
**P35213**	14-3-3 protein beta/alpha	758.4	1.12
**P62260**	14-3-3 protein epsilon	1450.7	1.16
**P68511**	14-3-3 protein eta	579.9	1.11
**P61983**	14-3-3 protein gamma	2087.9	1.13
**P68255**	14-3-3 protein theta	664.3	1.06
**P63102**	14-3-3 protein zeta/delta	1731.3	1.07
**P13233**	2',3'-cyclic-nucleotide 3'-phosphodiesterase	1391.4	-0.93
**P63039**	60 kDa heat shock protein, mitochondrial	609.7	1.09
**P19944**	60S acidic ribosomal protein P1	334.5	1.30
**P06761**	Endoplasmic reticulum chaperone BiP	454.0	1.11
**Q9ER34**	Aconitate hydratase, mitochondrial	1251.8	-0.92
**P68035**	Actin, alpha cardiac muscle 1	6416.6	-0.95
**P68136**	Actin, alpha skeletal muscle	6416.6	-0.92
**P62738**	Actin, aortic smooth muscle	6294.4	-0.93
**D3ZRN3**	Actin, beta-like 2	2448.4	-0.88
**P60711**	Actin, cytoplasmic 1	10898.3	-0.99
**P63259**	Actin, cytoplasmic 2	10898.3	-0.98
**P63269**	Actin, gamma-enteric smooth muscle	6294.4	-0.95
**B3DMA6**	Actin-like protein 9	102.1	-0.04
**P11030**	Acyl-CoA-binding protein	1463.2	1.15

**Q05962**	ADP/ATP translocase 1	601.2	1.07
**Q09073**	ADP/ATP translocase 2	487.4	1.08
**P04764**	Alpha-enolase	2858.3	1.04
**G3V8Q2**	Alpha-internexin	1172.1	1.08
**P37377**	Alpha-synuclein	326.4	1.17
**P13221**	Aspartate aminotransferase, cytoplasmic	1923.1	-0.98
**P00507**	Aspartate aminotransferase, mitochondrial	2025.1	1.06
**P19511**	ATP synthase F(0) complex subunit B1, mitochondrial	212.9	-0.44
**F1LP05**	ATP synthase subunit alpha	2920.7	1.12
**P15999**	ATP synthase subunit alpha, mitochondrial	3053.0	1.12
**G3V6D3**	ATP synthase subunit beta	8483.0	1.20
**P10719**	ATP synthase subunit beta, mitochondrial	8483.0	1.20
**P31399**	ATP synthase subunit d, mitochondrial	665.6	1.12
**P35434**	ATP synthase subunit delta, mitochondrial	314.8	1.17
**D4A133**	ATPase H+-transporting V1 subunit A	1680.6	-0.93
**G3V7L8**	ATPase, H+ transporting, V1 subunit E isoform 1, isoform CRA_a	177.7	1.26
**A0A0G2KBC7**	ATP-dependent 6-phosphofructokinase	136.8	-0.93
**P47858**	ATP-dependent 6-phosphofructokinase, muscle type	139.1	-0.90
**P15429**	Beta-enolase	883.6	1.05
**Q63754**	Beta-synuclein	2371.6	1.13
**G3V9G3**	Calcium/calmodulin-dependent protein kinase II, beta, isoform CRA_a	317.6	1.52
**F1LUE2**	Calcium/calmodulin-dependent protein kinase II, beta, isoform CRA_c	317.6	1.52
**P11275**	Calcium/calmodulin-dependent protein kinase type II subunit alpha	164.5	1.65
**P08413**	Calcium/calmodulin-dependent protein kinase type II subunit beta	317.6	1.52
**P15791**	Calcium/calmodulin-dependent protein kinase type II subunit delta	124.6	-0.91
**P11730**	Calcium/calmodulin-dependent protein kinase type II subunit gamma	185.3	1.65
**P18418**	Calreticulin	135.7	1.16
**Q5PPN4**	Carbonic anhydrase-related protein	135.8	1.15
**G3V936**	Citrate synthase	665.9	-0.93

**Q8VHF5**	Citrate synthase, mitochondrial	665.9	1.06
**P11442**	Clathrin heavy chain 1	174.4	1.06
**P45592**	Cofilin-1	3126.2	-0.95
**P07335**	Creatine kinase B-type	7714.4	1.02
**A0A0G2JVQ1**	Creatine kinase S-type, mitochondrial	131.4	1.07
**P25809**	Creatine kinase U-type, mitochondrial	593.6	-0.93
**Q9Z2F5**	C-terminal-binding protein 1	189.9	-0.68
**P32551**	Cytochrome b-c1 complex subunit 2, mitochondrial	401.5	1.13
**P20788**	Cytochrome b-c1 complex subunit Rieske, mitochondrial	1036.0	1.12
**D3ZD09**	Cytochrome c oxidase subunit	485.8	1.42
**P11240**	Cytochrome c oxidase subunit 5A, mitochondrial	1840.8	1.14
**P10818**	Cytochrome c oxidase subunit 6A1, mitochondrial	779.8	1.20
**D3ZSB0**	Cytochrome c oxidase subunit 6B1	485.8	1.39
**P48675**	Desmin	108.2	1.09
**Q6P6R2**	Dihydrolipoyl dehydrogenase, mitochondrial	210.0	1.32
**P08461**	Dihydrolipoyllysine-residue acetyltransferase component of pyruvate dehydrogenase complex, mitochondrial	104.1	1.07
**P11348**	Dihydropteridine reductase	626.1	1.14
**Q62950**	Dihydropyrimidinase-related protein 1	879.0	1.07
**P47942**	Dihydropyrimidinase-related protein 2	5459.2	-0.96
**Q62951**	Dihydropyrimidinase-related protein 4	520.6	-0.88
**Q08877**	Dynamin-3	119.3	-0.79
**M0R757**	Elongation factor 1-alpha	923.7	-0.96
**P62630**	Elongation factor 1-alpha 1	923.7	-0.96
**F1M6C2**	Elongation factor 1-alpha 1 pseudogene	891.6	-0.96
**P62632**	Elongation factor 1-alpha 2	1019.8	-0.97
**O35179**	Endophilin-A1	561.6	1.17
**Q9JMB3**	Erythrocyte membrane protein band 4.1-like 3	59.7	1.77
**Q5RKI1**	Eukaryotic initiation factor 4A-II	121.6	1.17

**A0A0G2K3Q6**	Fructose-bisphosphate aldolase	7008.0	-0.96
**P05065**	Fructose-bisphosphate aldolase A	2982.9	-0.95
**P09117**	Fructose-bisphosphate aldolase C	7008.0	-0.96
**Q5M964**	Fumarate hydratase 1	136.0	1.14
**P14408**	Fumarate hydratase, mitochondrial	180.1	1.15
**D4AA42**	G protein subunit alpha transducin 2	373.7	-0.51
**P07323**	Gamma-enolase	5573.8	-0.96
**P47819**	Glial fibrillary acidic protein	3967.4	1.14
**Q62669**	Globin a1	1266.1	1.22
**A0A0G2JSW3**	Globin a4	8209.0	1.05
**A0A0G2JSV6**	Globin c2	28416.9	-0.85
**Q6P6V0**	Glucose-6-phosphate isomerase	579.9	1.38
**P13264**	Glutaminase kidney isoform, mitochondrial	215.6	-0.60
**P04905**	Glutathione S-transferase Mu 1	238.2	-0.90
**P04797**	Glyceraldehyde-3-phosphate dehydrogenase	9155.3	1.13
**Q9ESV6**	Glyceraldehyde-3-phosphate dehydrogenase, testis-specific	468.4	1.26
**Q5EEY3**	GTP-binding protein G-alpha-i2 splice variant b	373.7	-0.49
**P54313**	Guanine nucleotide-binding protein G(I)/G(S)/G(T) subunit beta-2	319.4	-0.84
**P08753**	Guanine nucleotide-binding protein G(k) subunit alpha	373.7	-0.46
**G3V8E8**	Guanine nucleotide-binding protein G(olf) subunit alpha	373.7	-0.49
**P63095**	Guanine nucleotide-binding protein G(s) subunit alpha isoforms short	410.0	-0.49
**P29348**	Guanine nucleotide-binding protein G(t) subunit alpha-3	373.7	-0.49
**Q6Q7Y5**	Guanine nucleotide-binding protein subunit alpha-13	358.1	-0.48
**D4A752**	Guanine nucleotide-binding protein subunit beta-4	255.2	2.05
**P0DMW0**	Heat shock 70 kDa protein 1A	379.7	1.08
**P0DMW1**	Heat shock 70 kDa protein 1B	379.7	1.08
**P55063**	Heat shock 70 kDa protein 1-like	429.2	1.08
**P63018**	Heat shock cognate 71 kDa protein	3733.4	1.06
**Q5XHZ0**	Heat shock protein 75 kDa, mitochondrial	226.5	1.08

**P82995**	Heat shock protein HSP 90-alpha	884.6	-0.92
**P34058**	Heat shock protein HSP 90-beta	777.4	1.04
**P14659**	Heat shock-related 70 kDa protein 2	946.4	1.06
**P01946**	Hemoglobin subunit alpha-1/2	28416.9	-0.85
**P02091**	Hemoglobin subunit beta-1	8527.8	1.03
**A0A0G2JTW9**	Hemoglobin, beta adult major chain	1639.5	1.13
**Q6URK4**	Heterogeneous nuclear ribonucleoprotein A3	427.5	1.13
**P61980**	Heterogeneous nuclear ribonucleoprotein K	1160.4	1.14
**M0R9K1**	Heterogeneous nuclear ribonucleoprotein K-like	312.9	1.16
**F2Z3R2**	Heterogeneous nuclear ribonucleoprotein L	395.7	1.14
**A7VJC2**	Heterogeneous nuclear ribonucleoproteins A2/B1	475.1	1.09
**P62959**	Histidine triad nucleotide-binding protein 1	540.6	1.11
**D3ZWM5**	Histone H2B	5809.7	1.04
**Q00715**	Histone H2B type 1	5809.7	1.04
**D3ZK97**	Histone H3	336.5	-0.66
**Q6LED0**	Histone H3.1	561.6	-0.82
**P84245**	Histone H3.3	336.5	1.28
**Q9Z2X5**	Homer protein homolog 3	172.1	1.35
**Q99NA5**	Isocitrate dehydrogenase [NAD] subunit alpha, mitochondrial	174.9	1.40
**F1LNF7**	Isocitrate dehydrogenase [NAD] subunit, mitochondrial	174.9	1.42
**A0A0G2K4H7**	Keratin 78	206.0	-0.47
**P04642**	L-lactate dehydrogenase A chain	524.6	-0.66
**P30904**	Macrophage migration inhibitory factor	2371.8	1.06
**O88989**	Malate dehydrogenase, cytoplasmic	1468.1	1.07
**P43244**	Matrin-3	221.7	1.27
**P02688**	Myelin basic protein	12786.3	1.14
**P60203**	Myelin proteolipid protein	794.5	-0.92
**A0A0G2JWM2**	NAD-dependent protein deacetylase	110.6	-0.72
**Q5RJQ4**	NAD-dependent protein deacetylase sirtuin-2	110.6	-0.73

**Q561S0**	NADH dehydrogenase [ubiquinone] 1 alpha subcomplex subunit 10, mitochondrial	159.4	-0.70
**P19234**	NADH dehydrogenase [ubiquinone] flavoprotein 2, mitochondrial	392.3	1.22
**Q66HF1**	NADH-ubiquinone oxidoreductase 75 kDa subunit, mitochondrial	449.6	-0.89
**F1LQ81**	N-ethylmaleimide sensitive fusion protein, isoform CRA_b	130.8	1.09
**A0A0G2JWA1**	Neuroendocrine secretory protein 55	380.2	-0.49
**P16884**	Neurofilament heavy polypeptide	214.1	1.05
**P19527**	Neurofilament light polypeptide	1291.7	1.15
**P12839**	Neurofilament medium polypeptide	570.9	1.12
**Q05982**	Nucleoside diphosphate kinase A	593.1	1.12
**Q5BKC3**	Park7 protein	1040.0	1.13
**A0A0G2K1P0**	Peptidyl-prolyl cis-trans isomerase	4015.2	1.12
**P10111**	Peptidyl-prolyl cis-trans isomerase A	4015.2	1.11
**A0A0G2JSS8**	Peroxiredoxin 5, isoform CRA_c	596.7	1.17
**P35704**	Peroxiredoxin-2	414.5	1.08
**Q9R063**	Peroxiredoxin-5, mitochondrial	596.7	1.15
**O35244**	Peroxiredoxin-6	917.0	1.20
**P31044**	Phosphatidylethanolamine-binding protein 1	4527.1	1.20
**Q5XIV1**	Phosphoglycerate kinase	1487.7	-0.90
**P25113**	Phosphoglycerate mutase 1	2777.2	1.15
**O35264**	Platelet-activating factor acetylhydrolase IB subunit beta	334.6	1.09
**P54708**	Potassium-transporting ATPase alpha chain 2	148.6	-0.68
**A0A0H2UHM5**	Protein disulfide-isomerase	182.7	-0.75
**D3ZSW2**	Protein phosphatase 1, regulatory (inhibitor) subunit 1C	309.0	-0.43
**Q5XI34**	Protein phosphatase 2 (Formerly 2A), regulatory subunit A (PR 65), alpha isoform, isoform CRA_a	188.1	1.25
**O88767**	Protein/nucleic acid deglycase DJ-1	1154.6	1.12
**A0A140TAB9**	Protein-L-isoaspartate O-methyltransferase	1177.4	1.17
**P22062**	Protein-L-isoaspartate(D-aspartate) O-methyltransferase	1177.4	1.17
**D3ZXP8**	Purkinje cell protein 2	1113.3	1.14

**P52873**	Pyruvate carboxylase, mitochondrial	87.3	1.58
**D4A5G8**	Pyruvate dehydrogenase E1 component subunit alpha	78.3	1.82
**P26284**	Pyruvate dehydrogenase E1 component subunit alpha, somatic form, mitochondrial	78.3	1.80
**M0RD14**	Pyruvate kinase	1760.0	-0.96
**P12928**	Pyruvate kinase PKLR	965.7	-0.90
**P11980**	Pyruvate kinase PKM	2944.4	-0.94
**Q6AYT0**	Quinone oxidoreductase	149.7	-0.40
**P50398**	Rab GDP dissociation inhibitor alpha	1385.5	-0.85
**P50399**	Rab GDP dissociation inhibitor beta	809.4	-0.84
**G3V9A3**	RCG31390	287.6	1.08
**B5DFG5**	RCG53214, isoform CRA_d	123.8	1.19
**Q64548**	Reticulon-1	166.4	-0.79
**Q5XI73**	Rho GDP-dissociation inhibitor 1	266.8	1.30
**A0A1W2Q660**	Septin 14	59.9	1.19
**A0A0G2JUL7**	Septin 6 (Predicted), isoform CRA_b	62.0	1.23
**Q5PQK1**	Septin-10	59.9	1.20
**B3GNI6**	Septin-11	62.0	1.22
**Q9WVC0**	Septin-7	276.3	1.25
**A0A0G2K7T5**	Serine/threonine-protein phosphatase	98.4	1.80
**P63329**	Serine/threonine-protein phosphatase 2B catalytic subunit alpha isoform	158.5	1.93
**P20651**	Serine/threonine-protein phosphatase 2B catalytic subunit beta isoform	98.4	1.70
**P02770**	Serum albumin	525.5	1.04
**Q5EBB0**	Similar to 14-3-3 protein sigma	287.6	1.08
**Q498M9**	Similar to glyceraldehyde-3-phosphate dehydrogenase	1732.3	1.12
**F1LZI1**	Similar to heat shock protein 8	2908.4	1.06
**D4A3P7**	Similar to macrophage migration inhibitory factor	644.3	1.25
**F1LTZ6**	Similar to Macrophage migration inhibitory factor (MIF) (Delayed early response protein 6)	644.3	1.22
**G3V8S4**	Sodium/potassium-transporting ATPase subunit alpha	148.6	-0.68

**P06685**	Sodium/potassium-transporting ATPase subunit alpha-1	446.2	-0.66
**P06686**	Sodium/potassium-transporting ATPase subunit alpha-2	448.4	-0.63
**P06687**	Sodium/potassium-transporting ATPase subunit alpha-3	451.0	-0.65
**Q64541**	Sodium/potassium-transporting ATPase subunit alpha-4	253.7	-0.33
**A0A096MJI9**	Sodium/potassium-transporting ATPase subunit beta	724.8	-0.75
**P07340**	Sodium/potassium-transporting ATPase subunit beta-1	757.2	-0.75
**F1LX07**	Solute carrier family 25 member 12	547.2	-0.58
**P16086**	Spectrin alpha chain, non-erythrocytic 1	281.5	-0.88
**A0A0G2K8W9**	Spectrin beta chain	209.7	1.11
**Q63413**	Spliceosome RNA helicase Ddx39b	93.4	1.28
**P48721**	Stress-70 protein, mitochondrial	111.9	-0.90
**F1LM47**	Succinate--CoA ligase [ADP-forming] subunit beta, mitochondrial	104.4	-0.48
**P07632**	Superoxide dismutase [Cu-Zn]	830.4	1.20
**P07895**	Superoxide dismutase [Mn], mitochondrial	333.2	1.12
**P09951**	Synapsin-1	665.1	1.19
**G3V6M3**	Synaptotagmin II	149.9	-0.79
**P29101**	Synaptotagmin-2	149.9	-0.78
**A0A0G2K6I5**	Transgelin	181.4	1.13
**P37805**	Transgelin-3	281.5	1.11
**P50137**	Transketolase	211.4	1.38
**A0A0G2JWU1**	Triosephosphate isomerase	2758.5	-0.96
**A0A0H2UHM7**	Tubulin alpha chain	7058.4	-0.96
**P68370**	Tubulin alpha-1A chain	8555.0	-0.95
**Q6P9V9**	Tubulin alpha-1B chain	8116.3	-0.96
**Q6AYZ1**	Tubulin alpha-1C chain	7136.1	-0.96
**Q68FR8**	Tubulin alpha-3 chain	7146.8	-0.93
**Q5XIF6**	Tubulin alpha-4A chain	6951.7	-0.95
**Q6AY56**	Tubulin alpha-8 chain	4776.2	-0.94
**Q4QQV0**	Tubulin beta chain	7099.7	1.02

**P85108**	Tubulin beta-2A chain	11532.8	1.01
**Q3KRE8**	Tubulin beta-2B chain	11532.8	1.01
**P69897**	Tubulin beta-5 chain	11146.8	1.01
**Q00981**	Ubiquitin carboxyl-terminal hydrolase isozyme L1	747.7	1.20
**Q5U300**	Ubiquitin-like modifier-activating enzyme 1	133.2	1.65
**Q9QUL6**	Vesicle-fusing ATPase	130.8	1.25
**P31000**	Vimentin	294.2	1.11
**P62815**	V-type proton ATPase subunit B, brain isoform	352.8	-0.92
**Q6PCU2**	V-type proton ATPase subunit E 1	177.7	1.25
**Q8R2H0**	V-type proton ATPase subunit G	1130.5	-0.81

[a]Accesion ID from uniprot.org database; Negative and positive values of fold change represent down-regulation and up-regulation, respectively, in Pb group when compared to control group.

Table S2. Global proteomic profile of rats' cerebellum exposed to lead acetate in comparison to control group. List of proteins with exclusive expression in one of the groups.

			Fold change	
[a]Accession ID	Protein name description	PLGS Score	C	Pb
P26772	10 kDa heat shock protein, mitochondrial	645.0	-	+
Q5MYT7	2'-5'-oligoadenylate synthase 3	100.4	-	+
Q5XI78	2-oxoglutarate dehydrogenase, mitochondrial	89.8	+	-
O70351	3-hydroxyacyl-CoA dehydrogenase type-2	163.1	+	-
P46953	3-hydroxyanthranilate 3,4-dioxygenase	186.5	-	+
A0A0G2JSH2	3-hydroxybutyrate dehydrogenase, type 1, isoform CRA_a	105.9	+	-
Q91ZS3	45 kDa calcium-binding protein	232.9	-	+
G3V728	4-nitrophenylphosphatase domain and non-neuronal SNAP25-like protein homolog 1 (C. elegans), isoform CRA_b	240.6	-	+
P08909	5-hydroxytryptamine receptor 2C	166.4	-	+
B0ZTH9	6-phosphofructo-2-kinase/fructose-2, 6-bisphosphatase-4 transcript variant 3	130.9	-	+
P25114	6-phosphofructo-2-kinase/fructose-2,6-bisphosphatase 4	130.9	-	+
Q641Z7	Acid sphingomyelinase-like phosphodiesterase 3a	175.7	+	-
P49911	Acidic leucine-rich nuclear phosphoprotein 32 family member A	389.8	-	+
Q4V7C7	Actin-related protein 3	356.1	-	+
Q5XIK1	Actin-related protein T1	111.6	-	+
F8WG67	Acyl-CoA thioesterase 7, isoform CRA_a	149.2	+	-
Q9QYL8	Acyl-protein thioesterase 2	169.2	-	+
D3ZA74	ADAM metallopeptidase with thrombospondin type 1 motif, 14	98.3	+	-
A0A0G2K836	ADAM metallopeptidase with thrombospondin type 1 motif, 20	112.6	-	+
P10760	Adenosylhomocysteinase	82.0	-	+

P52481	Adenylyl cyclase-associated protein 2	110.2	-	+
Q66HA6	ADP-ribosylation factor-like protein 8B	215.2	-	+
P51635	Alcohol dehydrogenase [NADP(+)]	196.2	+	-
G3V7J0	Aldehyde dehydrogenase family 6, subfamily A1, isoform CRA_b	180.4	-	+
D3ZCV5	Aldehyde dehydrogenase, cytosolic 1	119.9	+	-
M0R8J2	Alpha-(1,3)-fucosyltransferase	91.0	-	+
Q5F2L1	Alpha-(1,3)-fucosyltransferase 10	92.6	-	+
P85515	Alpha-centractin	264.1	-	+
P54921	Alpha-soluble NSF attachment protein	175.1	+	-
D3ZG75	ALS2 C-terminal-like	110.6	+	-
P35433	Amidophosphoribosyltransferase	309.7	-	+
G3V846	Amino acid transporter	142.5	+	-
F1M7Q5	AMP deaminase	184.3	+	-
Q02356	AMP deaminase 2	95.1	-	+
A0A0G2JUT3	Androglobin	179.6	+	-
O35462	Angiopoietin-2	159.4	+	-
P62944	AP-2 complex subunit beta	247.3	-	+
D4AA14	Apoptosis-inducing factor, mitochondria-associated 2	305.7	-	+
B2RYJ7	ARP1 actin-related protein 1 homolog B	229.8	-	+
Q78E60	Aryl hydrocarbon receptor nuclear translocator 2	202.4	+	-
F1M9V0	Ataxin 7-like 1	169.9	+	-
Q6PDU7	ATP synthase subunit g, mitochondrial	479.6	-	+
Q6PCU0	ATP synthase subunit gamma	161.0	-	+
P35435	ATP synthase subunit gamma, mitochondrial	161.0	-	+
D3ZZS8	ATPase H+-transporting V1 subunit B1	91.5	+	-
E9PTI1	ATPase H+-transporting V1 subunit H	130.2	+	-
M0RA64	ATP-binding cassette subfamily B member 5	213.8	-	+
Q9QY44	ATP-binding cassette sub-family D member 2	112.6	-	+

A0A0A0MXY5	ATP-dependent 6-phosphofructokinase	107.6	-	+
P47860	ATP-dependent 6-phosphofructokinase, platelet type	87.1	-	+
Q9R1T1	Barrier-to-autointegration factor	339.2	+	-
D3ZU26	Basic helix-loop-helix family, member e2	170.8	+	-
Q10468	Beta-1,4 N-acetylgalactosaminyltransferase 1	245.2	-	+
Q02527	Beta-1,4-mannosyl-glycoprotein 4-beta-N-acetylglucosaminyltransferase	108.7	-	+
P85969	Beta-soluble NSF attachment protein	279.9	+	-
Q5HZA7	Bin1 protein	326.6	-	+
Q3ZB98	Breast carcinoma-amplified sequence 1 homolog	171.0	+	-
D4A8W8	BRF1 homolog, subunit of RNA polymerase III transcription initiation factor IIIB (S. cerevisiae) (Predicted), isoform CRA_a	96.3	-	+
Q5VLR5	BWK4	167.0	-	+
P27653	C-1-tetrahydrofolate synthase, cytoplasmic	202.2	+	-
F1MAH6	Cadherin 11	107.5	-	+
A0A0G2K7Z8	Cadherin 26	76.2	-	+
F1LMI3	Cadherin 3	96.9	-	+
P47728	Calretinin	183.8	-	+
P12368	cAMP-dependent protein kinase type II-alpha regulatory subunit	104.5	+	-
B0BNN3	Carbonic anhydrase 1	139.3	-	+
A2IBE2	Carbonic anhydrase 12	141.6	-	+
A0A0G2KB83	Carboxylic ester hydrolase	82.9	+	-
Q62761	Casein kinase I isoform gamma-1	60.5	-	+
G3V675	CASK-interacting protein CIP98, isoform CRA_b	101.0	-	+
Q5M7A7	CB1 cannabinoid receptor-interacting protein 1	115.1	+	-
Q8R1R5	CD99 antigen-like protein 2	245.9	+	-
Q1WIM3	Cell adhesion molecule 3	102.3	+	-
Q6AY41	Cell cycle control protein 50A	169.9	-	+
D4A9A3	Centromere protein V	111.8	+	-

D3Z9H1	Cerebellin 3 precursor	310.6	-	+
D3ZPV3	Circadian-associated repressor of transcription	133.4	+	-
Q05140	Clathrin coat assembly protein AP180	195.4	+	-
F1LVF5	Coiled-coil domain-containing 149	124.1	-	+
D3ZGD2	Coiled-coil domain-containing 24	428.4	-	+
D3ZBX9	Coiled-coil domain-containing 92	179.0	-	+
Q5PPN7	Coiled-coil domain-containing protein 51	92.3	-	+
F1M0M7	Collagen type XXVI alpha 1 chain	287.1	+	-
P63041	Complexin-1	206.3	-	+
Q63198	Contactin-1	101.3	-	+
Q0V8T3	Contactin-associated protein like 5-4	100.8	+	-
F1LMS4	Contactin-associated protein-like 5-3	100.8	+	-
D4A5C4	Corticotropin releasing hormone receptor 2, isoform CRA_a	240.5	-	+
P00564	Creatine kinase M-type	106.2	+	-
B1WC24	CTD small phosphatase 1	466.0	-	+
D4A5E4	Cyclin I	172.0	-	+
P39951	Cyclin-dependent kinase 1	209.6	-	+
F7EN52	Cyp46a1 protein	197.3	+	-
Q5M9I5	Cytochrome b-c1 complex subunit 6, mitochondrial	206.6	+	-
B2RYS2	Cytochrome b-c1 complex subunit 7	380.5	-	+
P00406	Cytochrome c oxidase subunit 2	109.9	-	+
D3ZFQ8	Cytochrome c-1	361.1	+	-
A0A0G2JWG7	Cytochrome P450, family 46, subfamily a, polypeptide 1	197.3	+	-
Q64559	Cytosolic acyl coenzyme A thioester hydrolase	149.2	+	-
Q2KN99	Cytospin-A	96.7	+	-
O08651	D-3-phosphoglycerate dehydrogenase	87.6	+	-
P29147	D-beta-hydroxybutyrate dehydrogenase, mitochondrial	105.9	+	-
A0A0G2K3F3	DC-STAMP domain-containing 2	176.6	-	+

A0A1W2Q637	D-dopachrome decarboxylase	404.7	-	+
A0A1W2Q6H6	Deleted in malignant brain tumors 1 protein	470.4	-	+
Q91Y53	Deoxynucleotidyltransferase terminal-interacting protein 1	126.6	+	-
D4A903	Dihydrodiol dehydrogenase	627.3	+	-
Q9JHU0	Dihydropyrimidinase-related protein 5	118.6	-	+
Q9EPB1	Dipeptidyl peptidase 2	199.3	-	+
Q63622	Disks large homolog 2	97.2	-	+
D3ZYP7	DNA polymerase gamma 2, accessory subunit	224.6	+	-
Q5M9H7	DnaJ (Hsp40) homolog, subfamily A, member 2	119.1	-	+
Q642C0	DnaJ homolog subfamily C member 8	201.3	-	+
D3ZR10	Doublecortin domain-containing protein 2	111.1	+	-
D4A9G5	Dpy-19-like C-mannosyltransferase 3	82.9	-	+
Q6AYH5	Dynactin subunit 2	135.4	-	+
M0R8N2	Dynein, axonemal, heavy chain 8	97.1	+	-
D3ZBM7	E3 ubiquitin-protein ligase HACE1	77.9	-	+
Q6MFZ5	E3 ubiquitin-protein ligase TRIM39	206.5	-	+
Q6P6T4	Echinoderm microtubule-associated protein-like 2	99.1	+	-
P84039	Ectonucleotide pyrophosphatase/phosphodiesterase family member 5	105.9	-	+
F1LTW9	EFR3 homolog B	160.0	+	-
F1LXD8	Elastin microfibril interfacer 2	204.9	-	+
P13803	Electron transfer flavoprotein subunit alpha, mitochondrial	196.8	-	+
Q68FR6	Elongation factor 1-gamma	188.0	-	+
P05197	Elongation factor 2	77.7	+	-
D4ACM1	Elongator complex protein 3	150.8	+	-
Q5PPJ9	Endophilin-B2	153.6	+	-
O08680	Ephrin type-A receptor 3	112.5	-	+

	Erythrocyte membrane protein band 4.1-like 2	106.9		
D3ZM69			-	+
G3V874	Erythrocyte membrane protein band 4.1-like 3	138.3	-	+
Q3B8Q2	Eukaryotic initiation factor 4A-III	139.5	-	+
B2RYN3	Eukaryotic translation elongation factor 1 epsilon 1	115.8	+	-
D3ZAZ0	Eukaryotic translation initiation factor 3 subunit M	150.4	+	-
Q6P3V8	Eukaryotic translation initiation factor 4A1	149.6	-	+
D4A895	Eva-1 homolog C	267.3	+	-
P24942	Excitatory amino acid transporter 1	145.6	+	-
B2GUZ5	F-actin-capping protein subunit alpha-1	235.7	-	+
M0R8F7	Family with sequence similarity 228, member B	91.6	+	-
D4A2L9	F-box and leucine-rich repeat protein 19	286.5	-	+
A0A0G2JSQ2	Flavin-containing monooxygenase	179.2	+	-
G3V6L1	Frizzled class receptor 6	92.9	+	-
Q66HT1	Fructose-bisphosphate aldolase	122.6	+	-
D3ZDZ7	Fucokinase	96.5	-	+
G3V8Z3	G patch domain and KOW motifs	300.8	+	-
P28473	Gamma-aminobutyric acid receptor subunit gamma-3	129.1	-	+
D3ZPV8	Gamma-glutamyl cyclotransferase	101.1	-	+
Q6QLN3	Glioma tumor suppressor candidate region gene 2	157.8	+	-
A0A0G2K1T0	Glutaminase kidney isoform, mitochondrial	215.6	+	-
Q5FWT5	Glutamyl-tRNA(Gln) amidotransferase subunit A, mitochondrial	432.9	-	+

M0R4L6	Glutamyl-tRNA(Gln) amidotransferase subunit B, mitochondrial	97.4	+	-
G3V8H3	Glutathione S-transferase	176.9	+	-
D3ZN47	Glycerol kinase 5 (putative)	261.0	-	+
P97564	Glycerol-3-phosphate acyltransferase 1, mitochondrial	208.5	-	+
O35077	Glycerol-3-phosphate dehydrogenase [NAD(+)], cytoplasmic	250.0	-	+
P00489	Glycogen phosphorylase, muscle form	2682.8	-	+
A0A0G2JXP1	Glycogenin-1	98.7	-	+
Q3ZU82	Golgin subfamily A member 5	101.4	+	-
P62828	GTP-binding nuclear protein Ran	126.7	+	-
Q9JHZ4	GRIP1-associated protein 1	30243.0	-	+
Q63942	GTP-binding protein Rab-3D	90.0	+	-
Q52KK4	H/ACA ribonucleoprotein complex non-core subunit NAF1	241.7	+	-
A0JPN1	H2.0-like homeobox protein	207.3	-	+
O88600	Heat shock 70 kDa protein 4	219.4	-	+
D3ZC55	Heat shock 70kDa protein 12A (Predicted), isoform CRA_a	101.7	+	-
D3ZPP6	HECT and RLD domain-containing E3 ubiquitin protein ligase 3	107.2	-	+
P04256	Heterogeneous nuclear ribonucleoprotein A1	287.4	-	+
Q62826	Heterogeneous nuclear ribonucleoprotein M	104.6	-	+
B0BNG5	Hif1an protein	109.9	+	-
A0A0H2UI35	Homer protein homolog 1	136.4	+	-
G3V683	Hypothetical LOC287938	354.0	-	+
M0R6E9	Immunoglobulin superfamily, member 7	331.1	-	+
M0R4G0	Immunoglobulin superfamily-containing leucine-rich repeat 2	121.9	-	+
P52296	Importin subunit beta-1	205.3	-	+
F1M5M3	Inactive serine/threonine-protein kinase TEX14	190.2	-	+
Q6AYK3	Inositol-3-phosphate synthase 1	252.2	-	+
D3ZN51	Integrin subunit alpha 9	99.0	+	-
Q6P6T3	Interferon gamma receptor 1	156.8	+	-

F1LZR4	Interleukin 31 receptor A	198.0	+	-
D3ZBV8	Interleukin-1 receptor-associated kinase 1-binding protein 1	135.6	+	-
P41565	Isocitrate dehydrogenase [NAD] subunit gamma 1, mitochondrial	265.9	+	-
Q5XIJ3	Isocitrate dehydrogenase [NAD] subunit, mitochondrial	262.8	+	-
A0A0G2JUF6	Isocitrate dehydrogenase [NADP]	109.4	-	+
P56574	Isocitrate dehydrogenase [NADP], mitochondrial	123.8	-	+
Q5BJY4	Josephin-1	290.0	+	-
F1M5A4	Katanin p60 ATPase-containing subunit A-like 2	104.0	+	-
Q5XIA9	Kelch domain-containing protein 8B	169.4	+	-
D3ZLT6	Kelch-like family member 31	93.6	+	-
A0A0G2JUR6	Keratin 78	82.6	-	+
G3V712	Keratin complex 2, basic, gene 7, isoform CRA_a	101.4	-	+
E9PSL8	Kinesin-like protein	75.9	+	-
Q62909	Kinesin-like protein KIF2C	90.1	-	+
A0A0G2K618	LARGE xylosyl- and glucuronyltransferase 1	141.3	+	-
B0BNG3	Lectin, mannose-binding 2	136.5	+	-
Q7TT51	Lengsin	86.6	+	-
D4A758	Leucine-rich repeat-containing 8 VRAC subunit B	302.1	-	+
P10867	L-gulonolactone oxidase	122.4	-	+
Q6AYF2	LIM and cysteine-rich domains 1	117.6	-	+
A0A0G2K8R3	LIM domain 7	100.7	+	-
F1M3U9	LisH domain-containing protein ARMC9	61.8	-	+
Q5RJS4	Listerin E3 ubiquitin protein ligase 1	311.4	-	+
Q924N5	Long-chain-fatty-acid--CoA ligase ACSBG1	66.9	-	+
D4A8G0	LSM12 homolog	134.6	+	-
D4A4U3	Magnesium-dependent phosphatase 1	294.5	+	-
D3ZAA9	MAGUK p55 subfamily member 2	268.7	-	+
A0A0G2K4C6	Malic enzyme	154.6	+	-

D4A980	MALT1 paracaspase	92.0	+	-
D3ZG00	Mediator complex subunit 30	164.0	+	-
A0A0G2KA58	Melanoma inhibitory activity 2	122.2	-	+
D3ZXB3	Mesoderm posterior 2 (Predicted)	98.8	-	+
D3ZPH3	Metastasis-associated 1 family, member 3	101.5	-	+
Q02253	Methylmalonate-semialdehyde dehydrogenase [acylating], mitochondrial	180.4	-	+
Q562C4	Methyltransferase-like protein 7B	236.6	+	-
D4A1Q2	Microtubule-associated protein	298.8	+	-
Q63560	Microtubule-associated protein 6	89.7	-	+
P19332	Microtubule-associated protein tau	298.8	+	-
Q6XVN8	Microtubule-associated proteins 1A/1B light chain 3A	284.4	+	-
A0A0G2K7P7	Mitochondrial carrier 2	307.0	-	+
Q4KM98	Mitochondrial fission factor	175.9	+	-
A0A0G2K2M2	Mitochondrial fission factor-like	175.9	+	-
Q925D6	Mitogen-activated protein kinase kinase 6	177.8	+	-
D3ZFL3	Mitogen-activated protein kinase kinase kinase 6	79.9	+	-
Q63562	Mitogen-activated protein kinase kinase kinase 8	88.1	+	-
G3V751	MRG domain-binding protein	124.4	+	-
O08839	Myc box-dependent-interacting protein 1	343.7	-	+
P20428	Myogenin	129.3	-	+
Q63518	Myosin-binding protein C, slow-type	178.6	-	+
P62775	Myotrophin	297.1	-	+
Q5PQT2	Myotubularin-related protein 3	94.4	-	+
A0A0G2KAQ5	Myozenin 2	241.8	+	-
Q769K2	N-acyl-phosphatidylethanolamine-hydrolyzing phospholipase D	331.8	+	-
Q1HCL7	NAD kinase 2, mitochondrial	86.3	-	+
F1M7T1	NADH dehydrogenase (ubiquinone) 1 beta subcomplex 4	215.8	+	-
D3ZCZ9	NADH dehydrogenase [ubiquinone] iron-sulfur protein 6, mitochondrial	284.5	+	-

P11661	NADH-ubiquinone oxidoreductase chain 5	95.4	+	-
A0A0G2K5W1	NCK-associated protein 5-like	72.3	-	+
D3ZD27	N-deacetylase and N-sulfotransferase 4	126.8	+	-
P13596	Neural cell adhesion molecule 1	91.8	-	+
D3ZWB7	Neuraminidase 4	140.5	+	-
P84076	Neuron-specific calcium-binding protein hippocalcin	304.0	+	-
F1LZB6	Nik-related kinase	195.7	-	+
D3ZYP9	NLR family, CARD domain-containing 3	73.8	-	+
D3ZE20	Nuclear factor of-activated T-cells 1	157.3	-	+
P61972	Nuclear transport factor 2	255.4	-	+
Q3B8Q1	Nucleolar RNA helicase 2	76.6	-	+
P13084	Nucleophosmin	255.6	+	-
G3V816	Nucleoside diphosphate kinase	152.1	+	-
D3ZC82	NUFIP2, FMR1-interacting protein 2	203.9	-	+
D4A840	Olfactory receptor	165.7	+	-
D3ZKR2	Olfactory receptor 1330	245.2	-	+
P04182	Ornithine aminotransferase, mitochondrial	197.1	+	-
Q5BK47	Oxysterol-binding protein	132.7	-	+
P0C548	Patatin-like phospholipase domain-containing protein 2	200.4	+	-
D3ZSF3	Peptidyl-prolyl cis-trans isomerase	127.5	+	-
Q62658	Peptidyl-prolyl cis-trans isomerase FKBP1A	550.0	-	+
Q8CJE2	Period circadian protein homolog 3	139.8	-	+
G3V7I0	Peroxiredoxin 3	159.4	+	-
Q5FVH2	Phospholipase D3	108.1	+	-
F1M208	Piezo-type mechanosensitive ion channel component	233.8	-	+
D3ZXL7	Piezo-type mechanosensitive ion channel component 2	267.3	-	+
Q6AYU5	Poly(rC)-binding protein 2	131.5	+	-
D3ZEH8	Polyamine-modulated factor 1	245.5	-	+

P19024	Potassium voltage-gated channel subfamily A member 5	93.4	+	-
D3ZEJ0	PR domain zinc finger protein 12	104.1	-	+
Q5M934	Probable tRNA pseudouridine synthase 1	92.8	-	+
D3ZFH6	Prohibitin, pseudogene 1	208.1	+	-
Q62849	Proprotein convertase subtilisin/kexin type 7	93.1	-	+
Q6P9V6	Proteasome subunit alpha type	112.4	+	-
P34064	Proteasome subunit alpha type-5	112.4	+	-
Q6AYM4	Protein DPCD	250.8	+	-
Q62902	Protein ERGIC-53	197.2	+	-
D3ZC07	Protein kinase N3	154.2	-	+
Q6J4I0	Protein phosphatase 1 regulatory subunit 1B	214.1	-	+
E9PTW7	Protein phosphatase 1, regulatory subunit 42	243.4	+	-
Q63945	Protein SET	154.2	-	+
M0R8Z9	Protocadherin alpha 2	174.2	-	+
M0RBC1	Protocadherin alpha-4	112.3	+	-
G3V9G0	Protocadherin beta 21	286.6	-	+
D4A455	Protocadherin gamma subfamily A, 10	83.6	+	-
A0A1B0GWS0	PTPRF-interacting protein alpha 1	88.9	+	-
P85973	Purine nucleoside phosphorylase	123.5	-	+
O35331	Pyridoxal kinase	399.2	-	+
Q5RKJ9	RAB10, member RAS oncogene family	90.0	+	-
A0A0G2K235	RAB1A, member RAS oncogene family	90.0	+	-
G3V6H0	RAB1B, member RAS oncogene family-like	90.0	+	-
A0A0G2JTT4	RAB30, member RAS oncogene family	90.0	+	-
D4A0G7	RAB37, member RAS oncogene family	90.0	+	-
D3ZZP2	RAB39, member RAS oncogene family (Predicted)	242.1	+	-
D4A7A8	Rab7b, member RAS oncogene family	145.9	-	+
A0A0G2K285	RALBP1 associated Eps domain containing protein 2	175.4	-	+

D3ZWW3	RAS protein activator-like 3	96.8	+	-
P35281	Ras-related protein Rab-10	90.0	+	-
P35284	Ras-related protein Rab-12	90.0	+	-
A0A1B0GWR3	Ras-related protein Rab-14	90.0	+	-
P35289	Ras-related protein Rab-15	132.1	+	-
Q6NYB7	Ras-related protein Rab-1A	90.0	+	-
P10536	Ras-related protein Rab-1B	90.0	+	-
P51156	Ras-related protein Rab-26	90.0	+	-
Q5U316	Ras-related protein Rab-35	90.0	+	-
Q63941	Ras-related protein Rab-3B	174.0	+	-
P62824	Ras-related protein Rab-3C	90.0	+	-
Q53B90	Ras-related protein Rab-43	90.0	+	-
P05714	Ras-related protein Rab-4A	90.0	+	-
P51146	Ras-related protein Rab-4B	90.0	+	-
P35280	Ras-related protein Rab-8A	90.0	+	-
P70550	Ras-related protein Rab-8B	90.0	+	-
D3ZTA4	RCG26849, isoform CRA_a	186.7	-	+
D3ZWT6	RCG31450	268.4	-	+
D4ADT0	RCG37011	175.7	+	-
Q6AXS1	RCG41520, isoform CRA_b	196.7	+	-
D3ZTS4	RCG42545, isoform CRA_b	174.2	+	-
D4AEK4	RCG49713	113.2	+	-
A0A096MJX6	RCG49877, isoform CRA_b	359.4	+	-
D4AE63	RCG55123, isoform CRA_a	207.4	-	+
D4A1N0	Receptor (chemosensory) transporter protein 3	97.6	+	-
A0A096MJ85	RGD1561796	359.4	+	-
A0A096MIZ0	Rhophilin, Rho GTPase binding protein 1 (Predicted), isoform CRA_b	114.9	+	-
A0A096MIY4	Rhophilin, Rho GTPase-binding protein 1	114.9	+	-

P29315	Ribonuclease inhibitor	138.5	+	-
Q5U2Q5	Ribonucleoside-diphosphate reductase	199.9	-	+
A0A1B0GWX1	Ribonucleotide reductase catalytic subunit M1	199.9	-	+
F1LXV0	Ribosomal protein S6 kinase	163.1	-	+
Q63531	Ribosomal protein S6 kinase alpha-1	163.1	-	+
D3ZRG6	Ring finger protein 6	125.8	+	-
D3ZND9	RIO kinase 3	149.0	-	+
A0A096MJ87	Septin 4	203.5	+	-
G3V8X9	Serine (or cysteine) peptidase inhibitor, clade A (alpha-1 antiproteinase, antitrypsin), member 16	87.8	+	-
D3ZKA0	Serine (Or cysteine) peptidase inhibitor, clade B (Ovalbumin), member 13 (Predicted)	78.3	-	+
A0A0G2QC06	Serotransferrin	365.3	-	+
B0BNE5	S-formylglutathione hydrolase	110.4	-	+
A0A096MJD8	SH2 domain-containing 5	305.7	-	+
P82450	Sialate O-acetylesterase	108.2	-	+
Q99PW3	Sialidase-1	152.8	+	-
Q4FZX7	Signal recognition particle receptor subunit beta	243.2	-	+
D4ACC5	Similar to 106 kDa O-GlcNAc transferase-interacting protein (Predicted), isoform CRA_a	121.8	-	+
F1M9V3	Similar to Alpha enolase (2-phospho-D-glycerate hydro-lyase)	105.9	-	+
D4AAN9	Similar to chromosome 6 open reading frame 52	219.8	-	+
F1M0M5	Similar to FLJ25323 protein	89.8	-	+
Q6AXV8	Similar to hypothetical protein 4930503F14	301.2	-	+
D3ZKZ0	Similar to Hypothetical protein MGC11690	317.1	+	-
F1M1I3	Similar to MIC2L1	207.9	-	+
D4A0K2	Similar to mitochondrial ribosomal protein S11	175.9	-	+
A0A0G2K896	Similar to RIKEN cDNA 1300017J02	135.8	+	-
A0A0G2JTV6	Similar to RIKEN cDNA 4931429L15	211.4	-	+
A0A0U1RRV3	Similar to RIKEN cDNA 4933402P03	167.8	+	-
A0A0G2JU14	Similar to RIKEN cDNA C430008C19	191.5	+	-

A0A0G2K628	SKI proto-oncogene	89.8	-	+
Q5M9H4	Sodium/potassium-transporting ATPase subunit beta	272.8	+	-
M0RCY3	Sodium/potassium-transporting ATPase subunit beta-1-interacting protein 2-like	373.4	-	+
P13638	Sodium/potassium-transporting ATPase subunit beta-2	272.8	+	-
A0A0G2K902	Sortilin-related VPS10 domain-containing receptor 2	192.7	+	-
A0A096MKE2	Sorting nexin 12	567.1	+	-
Q9JKU6	Spermatid perinuclear RNA-binding protein	101.6	-	+
Q5M877	SPRY domain-containing SOCS box protein 2	352.8	-	+
D4A7E9	Stomatin-like 1	213.6	+	-
P13086	Succinate--CoA ligase [ADP/GDP-forming] subunit alpha, mitochondrial	392.4	-	+
P51650	Succinate-semialdehyde dehydrogenase, mitochondrial	67.4	-	+
Q5BK78	Sulfatase-modifying factor 2	344.1	+	-
A0A0H2UI00	Sulfotransferase	231.0	+	-
G3V9R3	Sulfotransferase 1 family member D1	275.3	+	-
F1LVT6	SUMO1/sentrin-specific peptidase 1	62.0	-	+
D4A5W9	Synaptosomal-associated protein	140.0	+	-
P60881	Synaptosomal-associated protein 25	152.1	+	-
G3V7P1	Syntaxin-12	118.8	-	+
P61265	Syntaxin-1B	180.3	-	+
Q63635	Syntaxin-6	158.3	+	-
D4A6C9	Target of myb1-like 2 (Chicken) (Predicted), isoform CRA_a	253.7	-	+
A0A0G2K9L2	Target of myb1-like 2 membrane-trafficking protein	322.9	-	+
M0R6T1	TatD DNase domain-containing 1	143.5	-	+
Q5XIM9	T-complex protein 1 subunit beta	98.6	-	+
Q6P502	T-complex protein 1 subunit gamma	234.7	-	+
Q4V8I3	Tensin-4	105.6	+	-
Q4V8E7	Testis expressed gene 21	112.4	-	+
A0A0G2K6S3	Testis-expressed 21	118.4	-	+

A0A0G2JYJ8	Tetratricopeptide repeat domain 23-like	227.1	-	+
Q9Z0V6	Thioredoxin-dependent peroxide reductase, mitochondrial	159.4	+	-
Q5XIK2	Thioredoxin-related transmembrane protein 2	312.6	-	+
M0R402	Thioredoxin-related transmembrane protein 3	139.4	+	-
P24329	Thiosulfate sulfurtransferase	109.7	+	-
M0R6R9	Titin-like	642.8	-	+
M0R7K3	TNF alpha-induced protein 8-like 1	102.2	-	+
Q5XIC7	Transcription elongation factor A N-terminal and central domain-containing protein 2	119.3	-	+
Q63302	Transcription factor EC	373.0	-	+
P31232	Transgelin	133.0	-	+
M0RAT6	Transmembrane p24 trafficking protein 8	249.3	-	+
Q6AXS2	Transmembrane protein 252	561.1	-	+
Q64428	Trifunctional enzyme subunit alpha, mitochondrial	98.7	+	-
Q6P7B0	Tryptophan--tRNA ligase, cytoplasmic	103.8	+	-
D3ZLU5	TSPY-like 5	191.5	+	-
Q6PCT3	Tumor protein D54	120.0	+	-
Q4V888	Type 2 phosphatidylinositol 4,5-bisphosphate 4-phosphatase	127.2	+	-
Q5FVG7	Tyrosine-protein kinase	119.4	-	+
B5DF84	U3 small nucleolar RNA-associated protein 11	114.3	+	-
A0A0G2K8F7	Ubiquitin-conjugating enzyme E2A	420.3	-	+
M0R4X8	Ubiquitin-specific peptidase-like 1	174.2	+	-
M0R9B9	UBX domain-containing protein 7	103.5	+	-
D3ZCR9	UBX domain-containing protein 7-like	103.5	+	-
A0A0G2K4R2	UDP glycosyltransferase 3 family, polypeptide A2	81.7	-	+
Q6T5F2	UDP-glucuronosyltransferase	99.2	-	+
P20720	UDP-glucuronosyltransferase 1-2	99.2	-	+
Q4KM73	UMP-CMP kinase	174.7	-	+

D3ZDJ4	Unc-93 homolog B1 (C. elegans)	381.5	-	+
A0A0G2JW71	Unc-93 homolog B1, TLR-signaling regulator	381.5	-	+
A0A0G2JX05	Uridine-cytidine kinase	364.1	-	+
Q9QYG8	Uridine-cytidine kinase 2	364.1	-	+
Q63615	Vacuolar protein sorting-associated protein 33A	123.8	-	+
Q9ERB4	Versican core protein	76.5	-	+
Q9JI51	Vesicle transport through interaction with t-SNAREs homolog 1A	148.9	+	-
P62762	Visinin-like protein 1	189.5	+	-
D3ZE87	Vomeronasal 2 receptor, 30	128.2	-	+
Q5J3M1	Vomeronasal type-1 receptor	120.0	+	-
Q5M7T6	V-type proton ATPase subunit	96.0	-	+
Q5FVL0	V-type proton ATPase subunit d 2	122.0	+	-
Q810W9	Whirlin	101.0	-	+
Q5RJN8	YdjC chitooligosaccharide deacetylase homolog	279.7	-	+
E9PSN4	Zinc finger CCCH type-containing 13	287.4	-	+
Q642B9	Zinc finger protein 18	186.3	-	+
F1LSG4	Zinc finger protein 746	209.6	+	-

[a]Accesion ID from uniprot.org database; + sign represents exclusive regulation in the correspondent group, while – sign means absence of the protein in the correspondent group.
